# Untangling the complex interactions between turtle ants and their microbial partners

**DOI:** 10.1186/s42523-022-00223-7

**Published:** 2023-01-03

**Authors:** Manuela O. Ramalho, Corrie S. Moreau

**Affiliations:** 1 Department of Biology, West Chester University, 750 South Church Street, West Chester, PA 19383 USA; 2 Department of Entomology, Cornell University, Ithaca, NY 14853 USA; 3 Department of Ecology and Evolutionary Biology, Cornell University, Ithaca, NY 14853 USA

**Keywords:** Next-generation sequencing, Symbiont, Host-associated bacteria, Microbe

## Abstract

**Background:**

To understand the patterns of biodiversity it is important to consider symbiotic interactions as they can shape animal evolution. In several ant genera symbiotic interactions with microbial communities have been shown to have profound impacts for the host. For example, we know that for Camponotini the gut community can upgrade the host’s diet and is shaped by development and colony interactions. However, what is true for one ant group may not be true for another. For the microbial communities that have been examined across ants we see variation in the diversity, host factors that structure these communities, and the function these microbes provide for the host. In the herbivorous turtle ants (*Cephalotes*) their stable symbiotic interactions with gut bacteria have persisted for 50 million years with the gut bacteria synthesizing essential amino acids that are used by the host. Although we know the function for some of these turtle ant-associated bacteria there are still many open questions.

**Results:**

In the present study we examined microbial community diversity (16S rRNA and 18S rRNA amplicons) of more than 75 species of turtle ants across different geographic locations and in the context of the host’s phylogenetic history. Our results show (1) that belonging to a certain species and biogeographic regions are relevant to structuring the microbial community of turtle ants; (2) both bacterial and eukaryotic communities demonstrated correlations and cooccurrence within the ant host; (3) within the core bacterial community, Burkholderiaceae bacterial lineage were the only group that showed strong patterns of codiversification with the host, which is remarkable since the core bacterial community is stable and persistent.

**Conclusions:**

We concluded that for the turtle ants there is a diverse and evolutionarily stable core bacterial community, which leads to interesting questions about what microbial or host factors influence when these partner histories become evolutionarily intertwined.

**Supplementary Information:**

The online version contains supplementary material available at 10.1186/s42523-022-00223-7.

## Background

To fully understand global biodiversity, we must focus studies on hyper-diverse groups such as arthropods, fungi, and microbes, which have a very large number of species and disproportionately fewer scientific studies compared to charismatic megafauna [1]. Therefore, unraveling the diversity and distribution of microbial symbionts among insects is fundamental to understanding the biology and evolutionary history of the most diverse animal group on the planet. Among insects, ants are species rich and ecologically dominant in almost every terrestrial habitat [2]. Their evolutionary histories of more than 150 million years have been shaped by interactions with their microbial partners [3, 4], which have facilitated their dominance in several niches, such as the canopy of tropical forests [5, 6].

Previous studies have shown that ants engage in various symbiotic interactions with fungi [7, 8], other insects [9, 10] and also bacteria [4, 11, 12]. Focusing specifically on bacteria, many studies have shown that several species of ants rely on a diverse and functional symbiont community [4, 11, 13–17]. However, not all ant species rely on functional bacterial communities, i.e. *Crematogaster* [See 18].

Still, ants are a great system for examining interactions with their microbial communities, as they are eusocial insects, thus providing an ideal scenario to facilitate bacterial transfer between individuals from the same colony [19]. In addition, they also exhibit a variety of behaviors and ecologies that may predict the diversity of microbial communities. For example, several studies have shown the importance of the bacterial community in herbivorous ants and how they even exhibit changes in their intestinal morphology to house these microbial partners [4, 20–24]. Although ants with omnivorous and predatory habits tend to harbor fewer microbial partners [6, 18], they may still rely on bacterial communities ([15, 16, 25]. There are also species of ants that harbor more transient microbial partners, as is the case of species in the genus *Pheidole* [17] and *Atta sexdens* [26, 27]. Studies that explore diet preference and other ant behaviors and ecologies that may predict bacterial associations on a broad scale taking into account evolutionary time are still scarce (but see [4, 11, 28]) unlike in mammals and birds [29].

To date, few studies have explicitly examined ant microbial communities and their potential for codiversification with their ant hosts [15, 30–33] and turtle ants are a group that offer an excellent opportunity to explore this topic, as they harbor a diverse core bacterial community in their gut that has been diversifying with the host for about 50 million years [4, 31, 33–35]. Although the diet of the genus *Cephalotes* is not fully known, it is believed that their diet is primarily herbivorous, including sap, pollen, insect honeydew and extrafloral nectar [5, 36–38]. Turtle ants also feed on bird and mammal excrement for nutritional supplementation [39–42]. These bacterial communities have been shown to upgrade the diet of these hosts through the recycling of nutrients [35], which can also contribute to the thickness and formation of their exoskeleton [43].

Previous studies have shown that turtle ant bacterial communities can be structured between different colonies within the same species [34], and also within different gut compartments within species [14, 33, 44]. In addition, using 454 pyrosequencing, Sanders and collaborators [31] were able to identify the potential role of host phylogeny in structuring the entire intestinal bacterial community (16S rRNA) of 25 *Cephalotes* species. However, with an increased scale of sampling, the inclusion of a host phylogenetic tree with greater resolution generated through a reduced representation genome sequencing approach [45], and the continuous technological advances to both sample and analyze microbial communities this may provide us with greater insights into host specificity and the evolutionary histories of symbiotic microbes. Furthermore, as each bacterial lineage has an independent and complex history of how it was acquired by the host, studies that aim to understand each of these processes of acquisition of each bacterial lineage separately is crucial.

Therefore, with this study we intend to explore three questions in the diverse turtle ant microbiome: (1) What ecological factors structure the microbial communities of *Cephalotes*? (2) Are the bacterial and eukaryotic communities associated with turtle ants interacting? (3) Is the core bacterial community, which is stable and functional for the host codiversifying with the host when each bacterial lineage is analyzed independently? To achieve these goals, we used multiplex Illumina sequencing of 16S rRNA and 18S rRNA amplicons for more than 75 species of *Cephalotes* collected across the host’s geographic range. Our study provides new insights into this complex symbiotic interaction that turtle ants and their microbiomes have engaged for more than 50 million years.

## Results

In the present study we investigated microbial community diversity (bacterial with 16S rRNA and eukaryotic with 18S rRNA amplicons) of more than 75 species of turtle ants across their geographic range and in the context of the host’s phylogenetic history. Our bacterial 16S rRNA preliminary results found differences between pinned museum samples using a noninvasive method of DNA extraction (see Additional file 1) and for this reason these samples were excluded from subsequent analyses suggesting that pinned museum collections may not be appropriate for assessing insect-associated microbial communities. However, we did not find differences between samples of only the gaster/abdomen compared with the whole worker (Pseudo-F = 1.081, *p* value = 0.307) as previously found by Flynn et al. [33]. Our results also found no differences between the two different kits used in the present study (Pseudo-F = 1.068, *p* value = 0.322), so we kept samples from both in our library. After these initial filtrations and removing samples, our library contained 151 samples with 5,137,726 reads, varying with a maximum of 73,287 reads and a minimum of 2525 reads in the samples. Using BugBase, our data recovered more aerobic phenotypes than anaerobic in the 16S rRNA library (Additional file 2), which suggest turtle ant digestive tracts provide conditions that favor a certain type of bacteria.

Bacterial quantification (16S rRNA) was performed by qPCR and our results found significant differences between the bacterial communities for the host species (ANOVA, F = 4.031, *p* value = 3.9 e-10) and biogeographic regions (ANOVA, F = 2.422, *p* value = 0.022) included in the present study (Additional file 3). Exploring these qPCR results more deeply, we see that these significant results are driven by some specific host species and biogeographic regions. For species *C. adolphi* (decreasing from average), *C. atratus* and *C. unimaculatus* (both increasing from average), appear to be driving much of this difference, and for biogeographic regions our results indicate that the Antilles (increasing from average), is impacting this result. All results from these specific groupings can be found highlighted in Additional file 4.

**Fig. 1:**
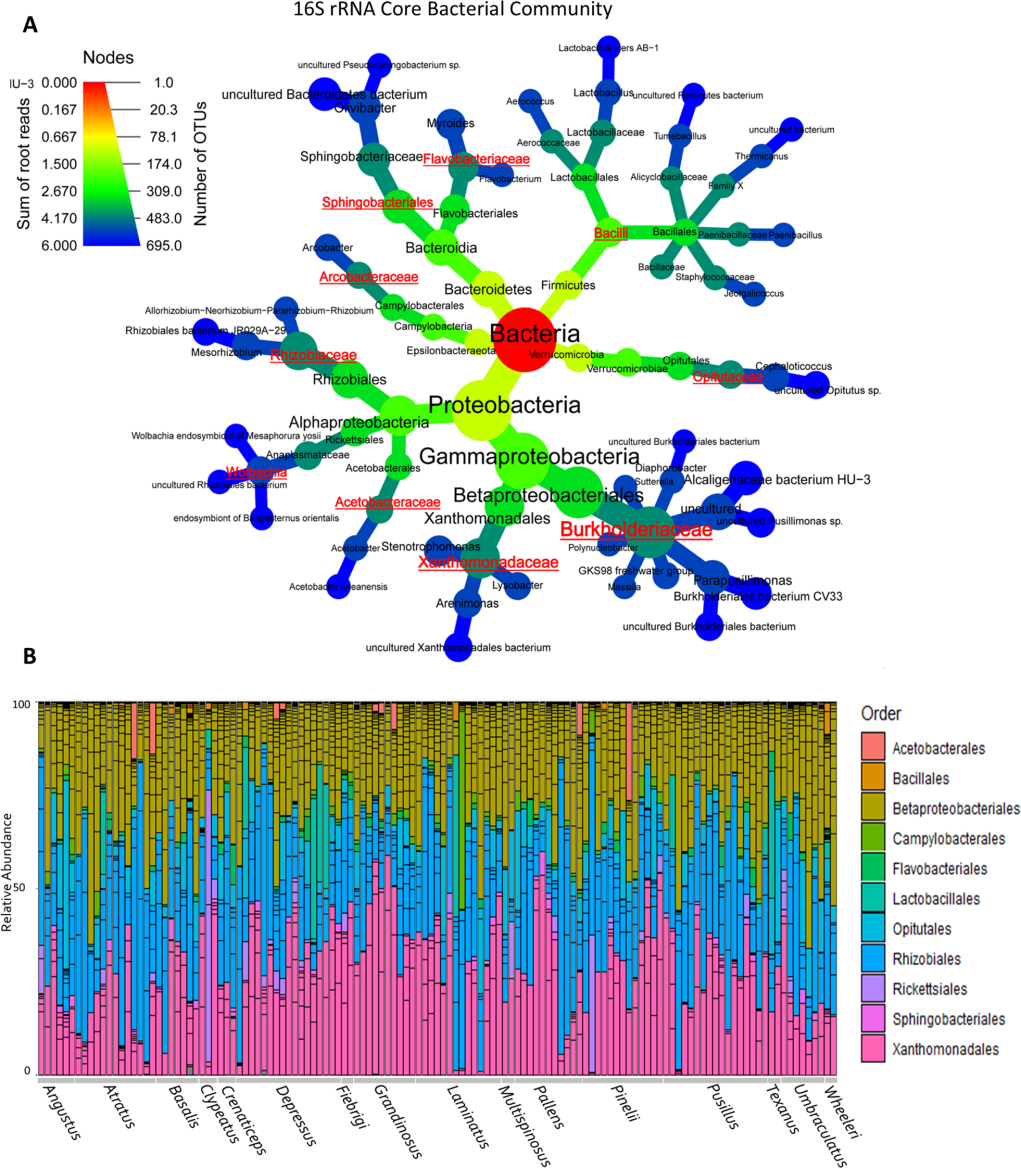
Bacterial communities (16S rRNA) associated with turtle ants. **A** Heat tree phylogeny highlighting in red text the core bacterial community of *Cephalotes* from the present study. **B** Stacked barplots show the relative abundance of the bacterial order composition (16S rRNA) of each individual sample (column) with the host species group denoted along the bottom

**Fig. 2:**
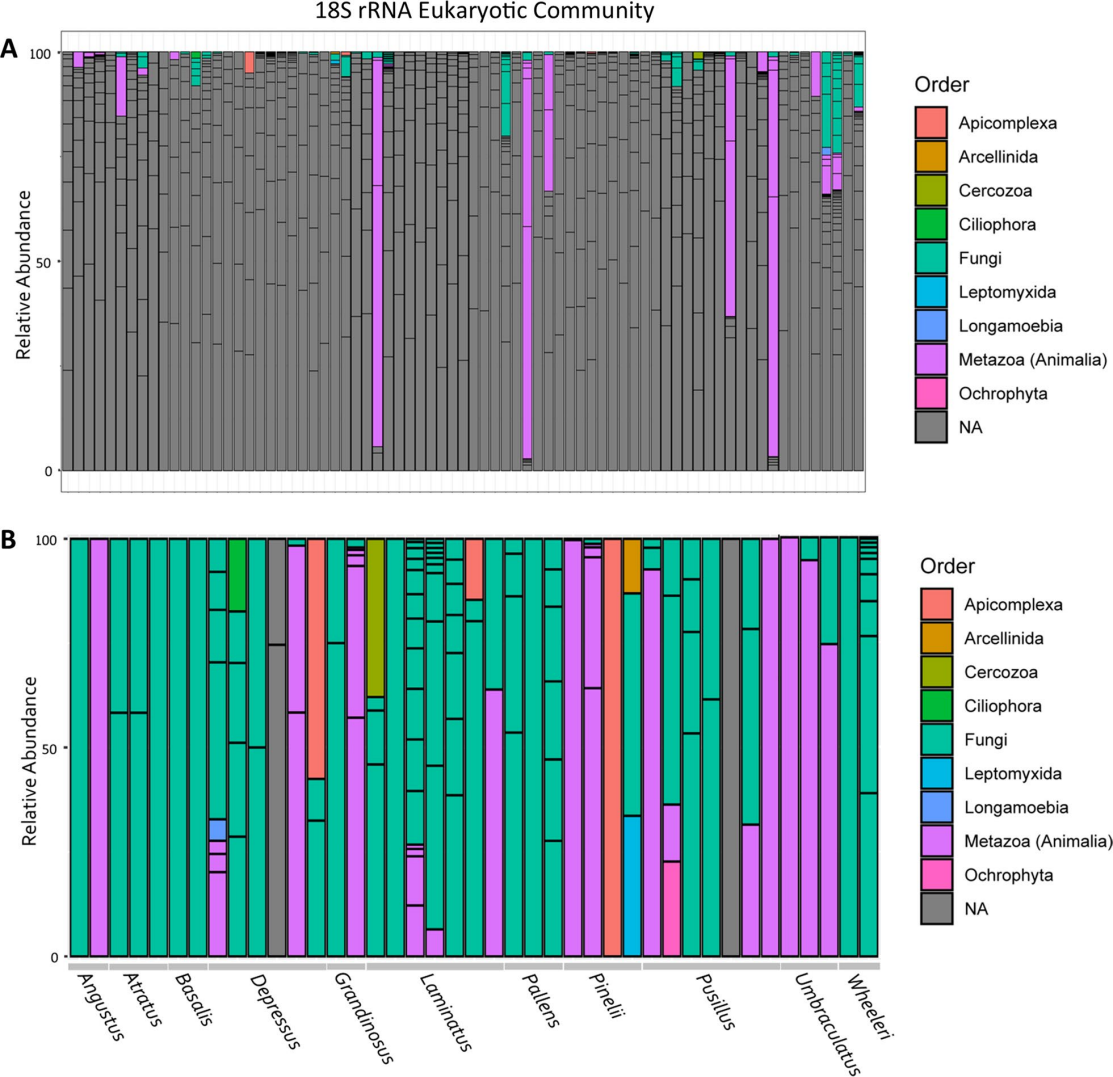
Microbial eukaryotic communities (18S rRNA) associated with turtle ants. **A** Stacked barplot showing the relative abundance of eukaryote order composition (18S rRNA) of each individual sample (column). Unclassified orders are represented in gray. **B** Filtered stacked barplots showing the relative abundance eukaryote order composition (18S rRNA) of each individual sample (column) with the host species group denoted along the bottom

We define the core bacterial community of *Cephalotes* samples as ASVs present in at least 50% of all individuals since we are looking across species that span more than 50 million years of host evolution [46]. Our core microbiome analyses sought to find shared bacterial members among turtle ant’s samples. By identifying and applying core bacterial communities in microbial ecology studies is crucial to target ASVs that may play key roles in host-microbe interaction [47]. In addition, we examined the core community at several taxonomic levels with the PhyloCore program, whose algorithm identifies essential taxa through microbial phylogeny and presence data [48], and our results can be viewed in the heat tree, with the core taxa highlighted in red text (Fig. 1A). This means that all ASVs that had the taxonomic level equal to or below those highlighted in red text were included in the subsequent analyses and are displayed in Fig. 1B. For the 18S rRNA library it was not possible to identify the presence of a core community, because they were more variable and not stable. Our 18S rRNA analysis was also unable to reach lower taxonomic levels since most ASVs were only assigned to the Eukaryote level, highlighted in gray bars in Fig. 2A. The reason for this is because the 18S rRNA database is not as well resolved or populated as the bacterial community database. For subsequent analyses, we removed these taxa only assigned to Eukaryote, and continued with the 43 samples assigned to lower taxonomic ranks. The diversity of these remaining samples can be seen in Fig. 2B.

### Alpha diversity

Overall, the core bacterial community (16S rRNA) was composed of 29% Burkholderiaceae, 28% Xanthomonadaceae, 26% Rhizobiaceae, followed by 7% Opitutaceae, 2% Rickettsiaceae *(Wolbachia*), 2% Lactobacillaceae (*Lactobacillus*), and others in smaller abundance (See Fig. 1B). For our 18S rRNA library, after removing ASVs returned as Eukaryote, we still recover 63% Metazoa, 36% Fungi, and others in smaller abundance (See Fig. 2B).

Overall, our data show high bacterial alpha diversity within *Cephalotes* with Shannon and Simpson metrics (Additional file 5) when compared with *Daceton armigerum*, *Camponotus* spp. and Ponerine ants workers [25, 49–51]. In addition, our data indicate that this high alpha diversity occurs in all biogeographic regions and in general for the different species, except for a few samples. However, we found host species-based differences in alpha diversity analysis in the full dataset (Kruskal–Wallis—all groups, H = 78.650, *p* value = 0.03). Still, when we explore these pairwise results, we see that only a few combinations of species are driving these results. Those species that showed significant results (< 0.05) are highlighted in Additional file 6. This result was also explored for biogeographic region-based differences in alpha diversity and our full dataset did not show significant results (Kruskal–Wallis—all groups, H = 6.180, *p* value = 0.518).

To explore these results more deeply, we investigated whether any core bacteria exhibit specificity to a particular species or biogeographic region, or whether the distribution of ASVs is more widespread. Our results presented in Additional file 7 show this generalized pattern, therefore there is no specificity. Still, four main ASV bacterial orders were recurrent in terms of abundance in our study, and they are the Xanthomonadales, Rhizobiales, Opitutales and Betaproteobacteriales (which includes Burkholderiaceae) and this can be seen both in the cluster for species (Additional file 7A) as well as for the different biogeographic regions (Additional file 7B).

Considering our eukaryotic community data associated with turtle ants, we did not find species-based differences in alpha diversity in the full dataset (Kruskal–Wallis—all groups, H = 27.043, *p* value = 0.254) or in biogeographic region-based comparisons (Kruskal–Wallis—all groups, H = 4427, *p* value = 0.729). We also explored if any microbial eukaryotes exhibit specificity to a particular species or biogeographic region. However due to the low taxonomic resolution of the 18S rRNA database, a large part of our data was removed (those who were only identifed as Eukaryote). Therefore, we cannot thoroughly investigate this question (Additional file 8).

### Beta diversity

**Fig. 3:**
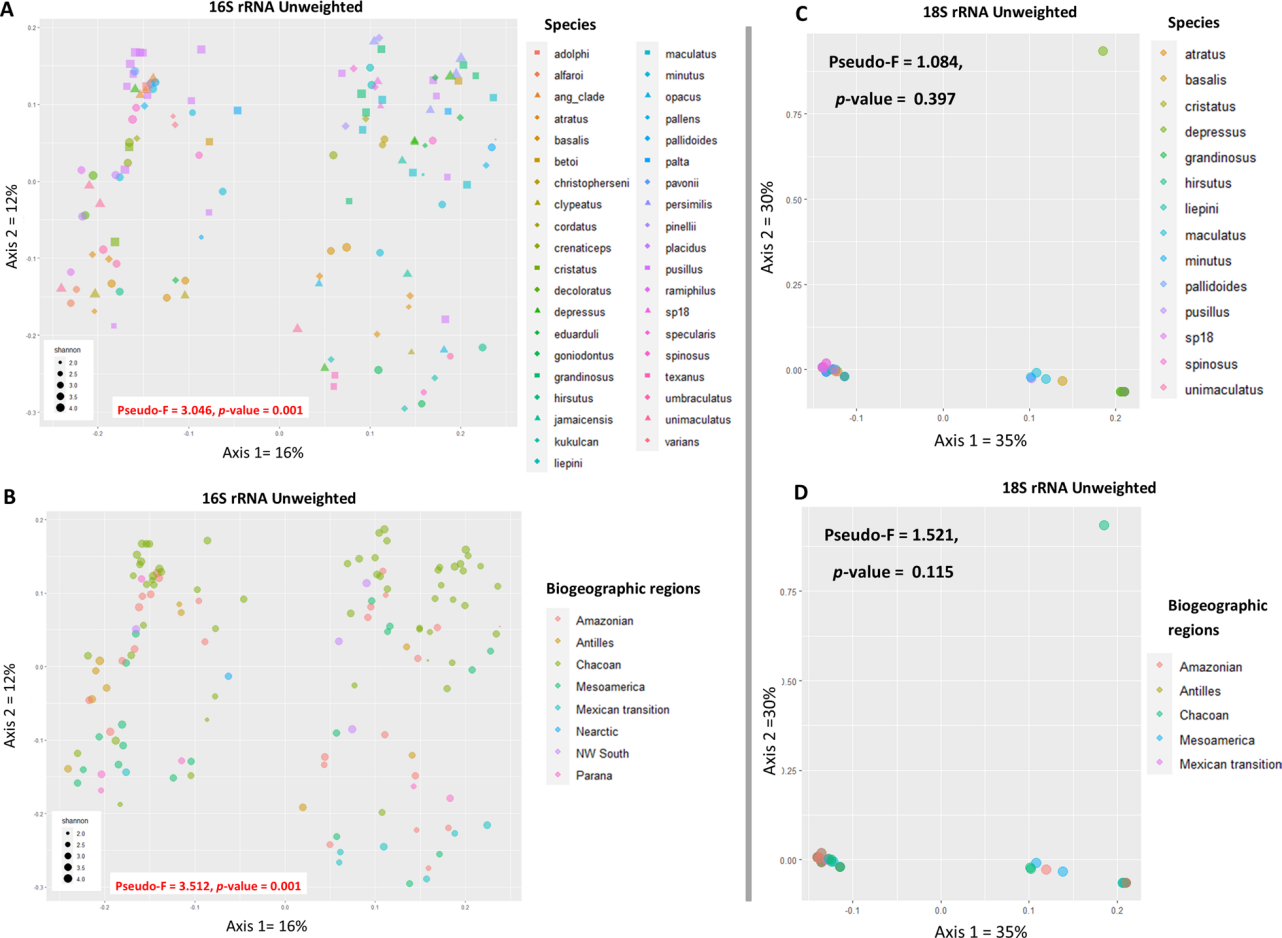
Impact of composition and abundance of the bacterial community (16S rRNA) and of microbial eukaryotes (18S rRNA) associated with *Cephalotes* (beta diversity) belonging to different host species and biogeographic regions. **A** PCoA of bacterial community (unweighted unifrac distance matrix) of different *Cephalotes* species. **B** PCoA of bacterial community (unweighted unifrac distance matrix) of different biogeographic regions. **C** PCoA of microbial eukaryotic communities (unweighted unifrac distance matrix) of different *Cephalotes* species. **D** PCoA of microbial eukaryotic communities (unweighted unifrac distance matrix) of different biogeographic regions

Our beta diversity analyses sought to assess how species identity and biogeographic region influence the composition (unweighted unifrac distance) and abundance (unweighted unifrac distance) in both bacterial (16S rRNA) and eukaryotic (18S rRNA) communities (Fig. 3) of turtle ants. The two categories that seem to impact the composition and abundance of bacterial communities (16S rRNA) are belonging to different host species of *Cephalotes* (unweighted unifrac, Pseudo-F = 3.046, *p* value = 0.001, weighted unifrac, Pseudo-F = 1.850, *p* value = 0.001) and different biogeographic regions (unweighted unifrac, Pseudo-F = 3.512, *p* value = 0.001, weighted unifrac, Pseudo-F = 1.842, *p* value = 0.004). The unweighted unifrac distance results can be seen in Fig. 3A, B. We also explored the non-metric multidimensional scaling (NMDS) ordination with the Bray Curtis metric of the different species and biogeographic regions that considers both the composition and abundance of *Cephalotes* samples present in this study and found both categories can structure bacterial communities (Additional file 9).

For the microbial eukaryotes (18S rRNA) host species has no impact on both composition and abundance (unweighted unifrac, Pseudo-F = 1.084, *p* value = 0.397, weighted unifrac, Pseudo-F = 1.121, *p* value = 0.347). Also, when we explore the different biogeographic regions, the same pattern was identified with no impact on both composition and abundance (unweighted unifrac, Pseudo-F = 1.521, *p* value = 0.115, weighted unifrac, Pseudo-F = 1.374, *p* value = 0.130) (see unweighted unifrac results in Fig. 3C, D).

**Fig. 4:**
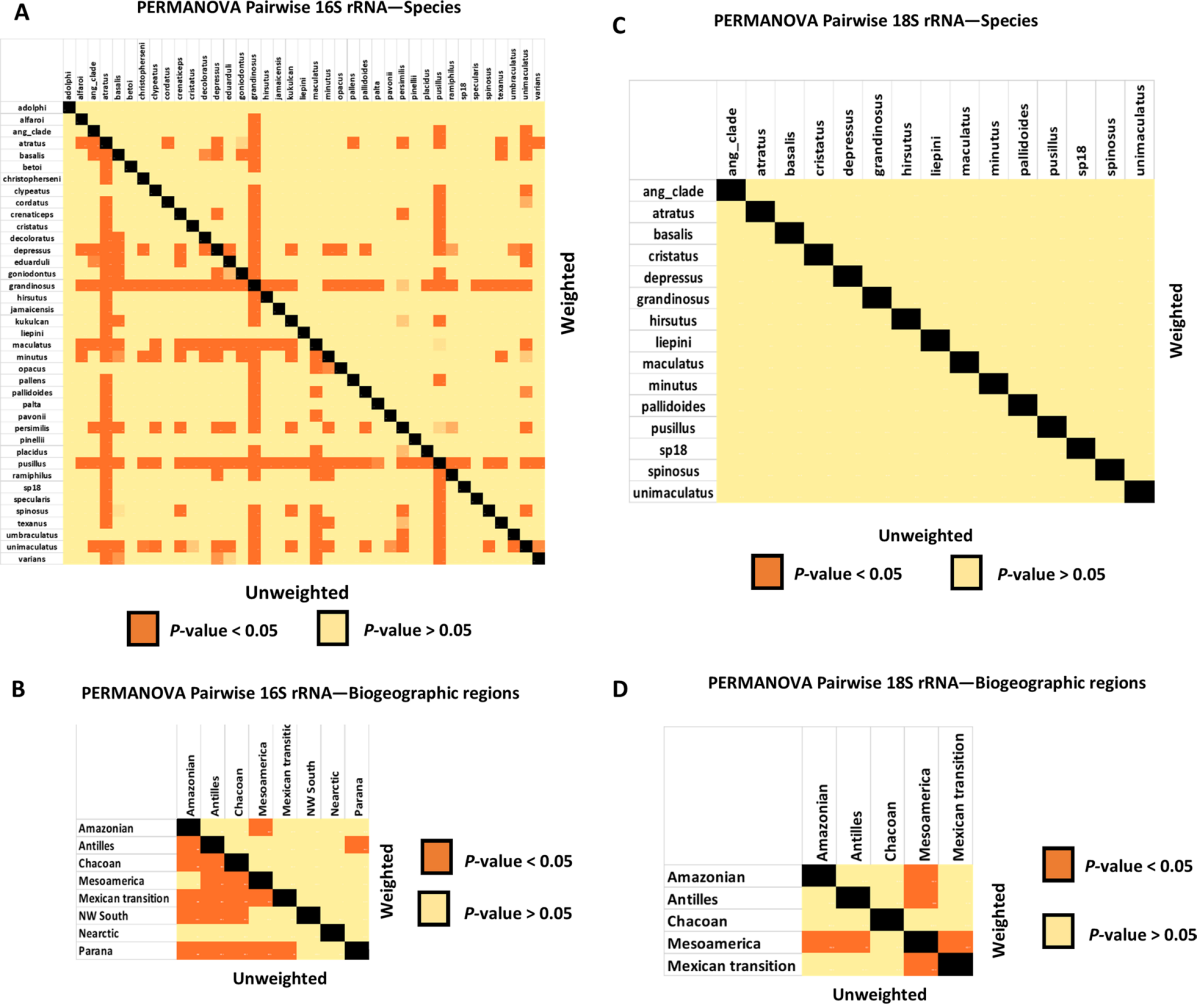
Pairwise PERMANOVA results of bacteria and eukaryotic microbial communities. **A** Unweighted and weighted unifrac distances of bacterial communities highlight in orange the groupings of host species that are impacting the results (*p *value < 0.05). **B** Unweighted and weighted unifrac distances of bacterial communities highlight in orange the groupings of biogeographic regions that are impacting the results (*p* value < 0.05). **C** Unweighted and weighted unifrac distances of microbial eukaryotes highlighting in orange the groupings of host species that are impacting the results (*p* value < 0.05). **D** Unweighted and weighted unifrac distances of microbial eukaryotes highlighting in orange the groupings of biogeographic regions that are impacting the results (*p* value < 0.05)

In addition, if we explore the potential clusters responsible for these significant results in the pairwise PERMANOVA results highlighted in orange in Fig. 4A, B of the bacteria and Fig. 4C, D of the eukaryotes (clusters *p *value < 0.05). We see that for the groupings of species, both the composition and the abundance of bacterial communities seems to be significant overall for species *C. grandinosus* and *C. pusillus*, but considering only the composition, we find overall differences between *C. atratus* and *C. maculatus* (Fig. 4A). In terms of the composition of the bacterial community of the biogeographic regions, in general, we found differences in practically all groups, except for the Nearctic region. In addition, we found no differences regarding the abundance of bacterial communities in the different biogeographic regions (Fig. 4B). Overall, for the eukaryotic microbial communities in the groupings of species we did not find any significant clustering in in composition and abundance (Fig. 4C). For biogeographic regions we found differences in composition and abundance for the Mesoamerica region. (Fig. 4D).

### Correlation between bacterial (16S rRNA) and eukaryote (18S rRNA) communities

**Fig. 5:**
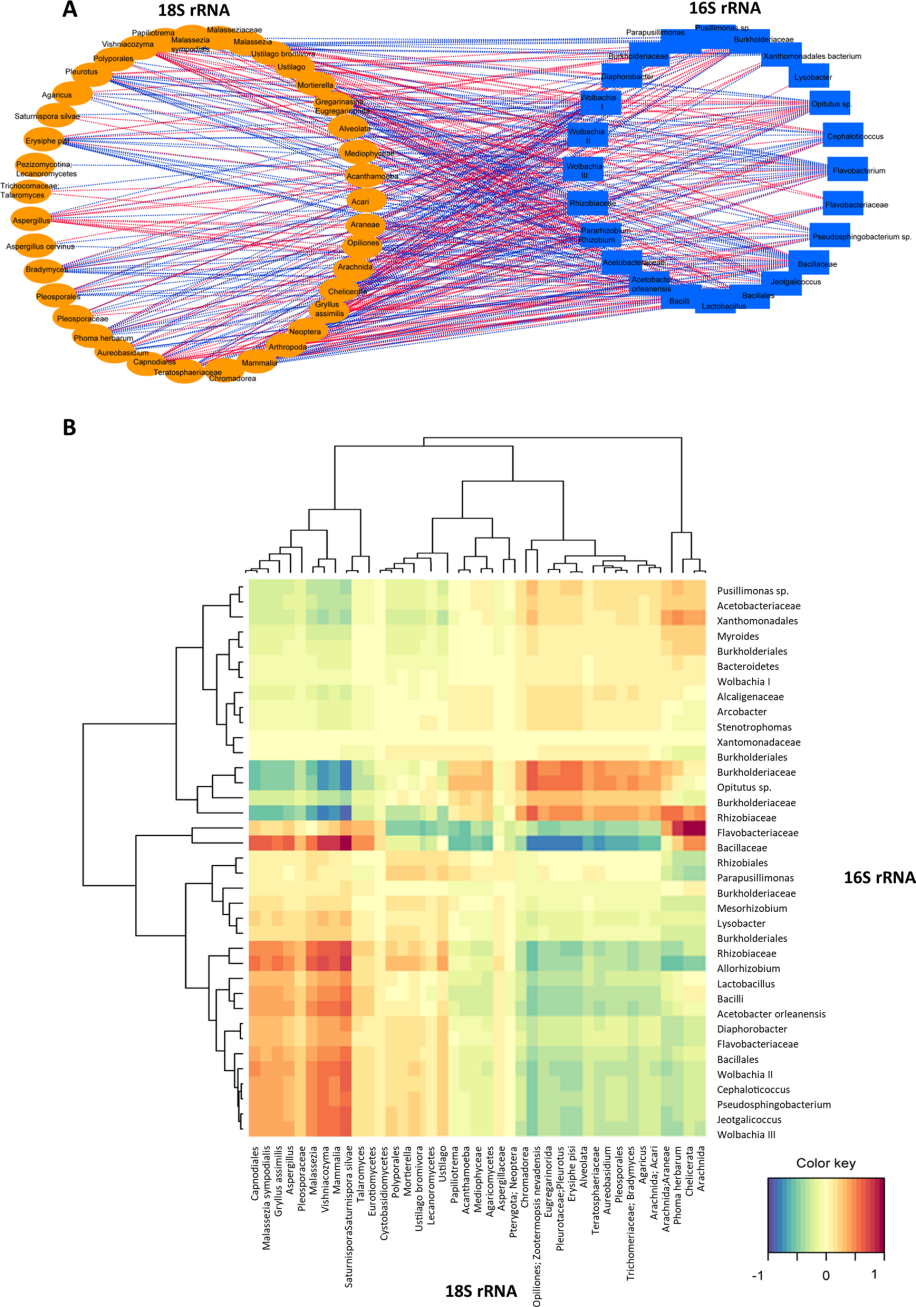
Correlation between bacterial and eukaryotic communities of *Cephalotes* hosts. **A** Regularized Generalized Canonical Correlation Analysis (rGCCA) correlation network between bacterial (16S rRNA) and eukaryotes (18S rRNA) communities associated with *Cephalotes* samples. Positive correlations are represented by red edges, and negative correlations by blue edges. **B** Heatmap indicating the positive (red) and negative (blue) correlations between the bacterial and eukaryotic communities of *Cephalotes* samples. The bacterial and eukaryotic ASVs were grouped according to the similarity of the correlations, as seen with the trees

Our analysis revealed 359 significant correlations (*p *value less than 0.05) between 16S rRNA and 18S rRNA ASVs associated with the *Cephalotes* samples, with 172 interactions identified as positive correlations and 187 as negative correlations as can be seen through the network structure in Fig. 5A. The positive (red) and negative (blue) correlations of bacteria and eukaryotes in the community can be identified in pairs as seen in the heatmap (Fig. 5B). These results indicate that some of the bacterial diversity is likely from their eukaryote microbes and should be further investigated to be fully understood. However, some of these interactions, especially of bacteria, may be occurring due to ant microbe interactions, which we further investigated in the present study in the co-diversification section below.

### Coevolutionary patterns of the core bacterial lineages of the turtle ants

**Fig. 6:**
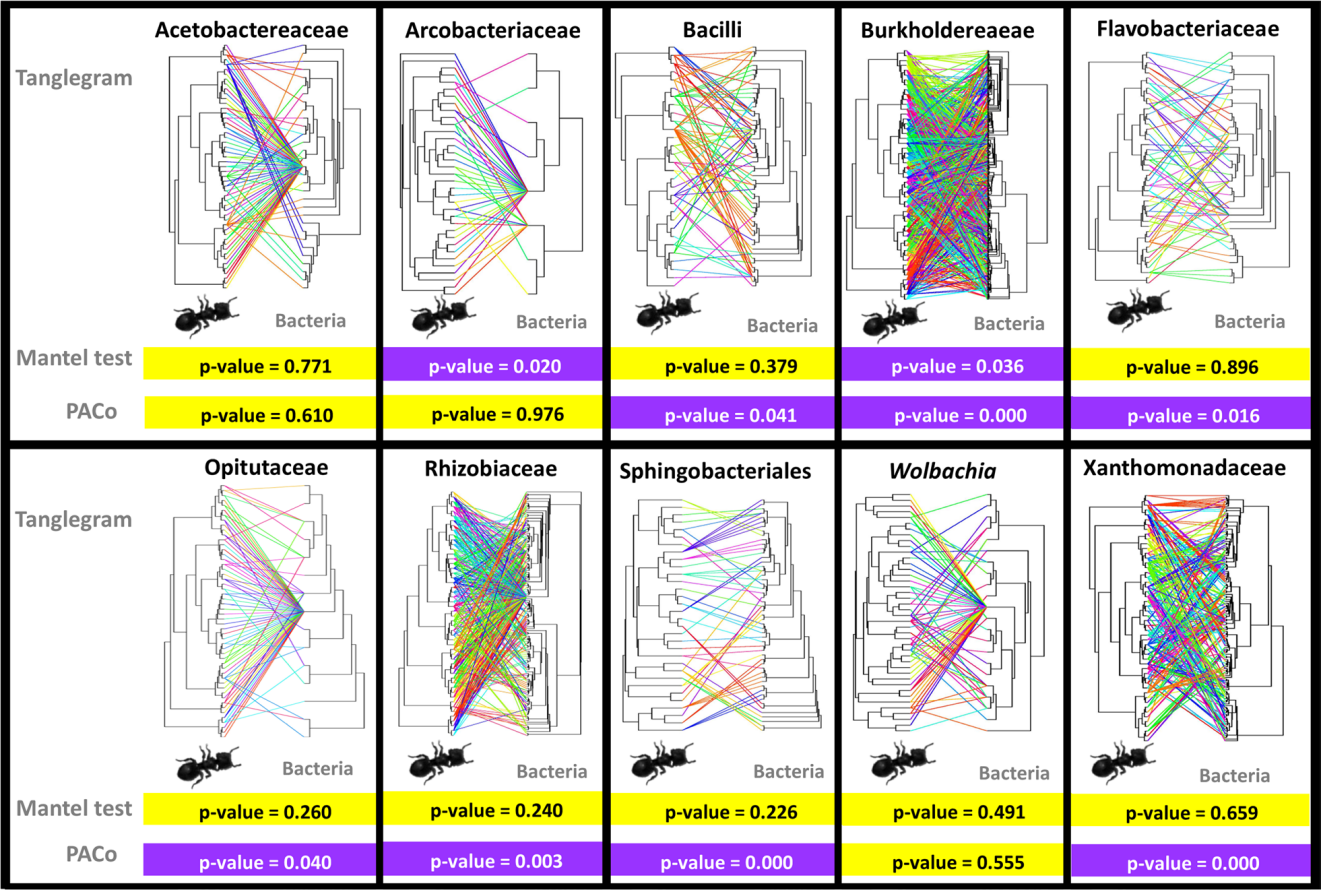
Exploring the coevolution signal of each core bacterial lineage associated with *Cephalotes.* Note that in addition to visualizing the bacteria occurrence in the host through a tanglegram, two statistical tests are applied to explore the coevolutionary signal of the bacterial communities and turtle ant evolutionary history: Mantel test and PACo analysis. *P* value less than 0.05 suggesting codiversification are highlighted in purple, and greater than 0.05 highlighted in yellow

This analysis was performed with the most recent and robust phylogenomic tree (UCE) for the genus *Cephalotes* [45]. Each core bacterial community associated with *Cephalotes* turtle ants was analyzed separately to understand the evolutionary history of these associations with the Mantel test and PACo (Procrustean Approach to Cophylogeny) analyses (Fig. 6). Comparing the two results, the Mantel tests show more conserved results for our data than the PACo analysis. Of all the bacteria analyzed, the Burkholderiaceae ASVs were the only group that showed strong patterns of coevolution with their host *Cephalotes* species for both the Mantel test and PACo analyses. The Arcobacteriaceae also shows significant Mantel test results, but when examining the tanglegram and the PACo analysis we see that this result is probably being impacted by having few representative ASVs associated with the core microbiome of *Cephalotes* species and should be further studied. Two other groups of bacteria that did not show a sign of codiversification with turtle ants by either of the two methods are *Wolbachia* and Acetobacteriaceae (Fig. 6), while the remaining show varied support for codiversification with their turtle ant hosts between statistical methods. As there is a discussion in the literature about the use of the Mantel test in some cases in phylogenetic comparative analyses (see [52]), we decided to show both statistical results, especially highlighting the differing results, so that future studies may consider using at least another test besides Mantel for a more reliable analysis. If we exclude the results from the Mantel test, we find that several other core bacterial groups may be codiversifying with their turtle ant hosts including Bacilli, Flavobacteriaceae, Opitutaceae, Rhizobiaceae, Sphingobacteriales, and Xanthomonadaceae.

## Discussion

Investigating host-associated microbial communities can reveal insights into the ecological and evolutionary success of the host [53, 54]. Insects are one of the groups of animals with the greatest diversity and dominate terrestrial ecosystems [2], and it is believed that this success has been partially achieved due to their microbial partners [55–57]. Within the microbiome there may be mutualistic, parasitic and commensal species associated with a host [58–60], however there are still few studies that explore symbiotic interactions across prokaryotic and eukaryotic microbes in invertebrate hosts [25, 53]. These symbionts are likely transmitted in different ways, with or without sharing coevolutionary stories with their host. Microbial communities are extremely diverse, and they occur in a variety of locations within the body and tissues of the host insect [55–57].

Within the Formicidae ant family it is no different and growing evidence suggests that the partnership with microbes was fundamental for ants to reach new niches [2, 4]. Many studies with ants have shown that they engage in diverse symbiotic interactions with diverse factors structuring these microbial communities [4, 16, 17, 26, 31, 32, 34, 35, 44, 49, 50, 61]. However, the majority of these studies focus only on bacteria, leaving underexplored other microbial partners (with few exceptions [8, 25]).

The core bacterial community results from this study corroborate previous work on turtle ants, emphasizing that associated bacteria are highly conserved [31, 33–35]. As in these studies, the core bacteria that we found were Burkholderiales, Opitutales, Pseudomonadales, Rhizobiales, and Xanthomonadales, and in addition to those, we also recovered other bacterial groups in high abundance across host samples such as Bacilli, Sphingobacteriales, Flavobacteriaceae, Arcobacteraceae, Acetobacteraceae and *Wolbachia.* This pattern of conserved bacterial community has already been observed by other insects such as the social bee [62] and also in ants of the Camponotini tribe [28, 32, 49, 50, 63]. As in Camponotini-*Blochmannia* interactions, the core bacterial community of *Cephalotes* provides fundamental functions for the host by contributing to nutritional upgrading with nitrogen recycling and synthesis of essential and non-essential amino acids [35]. Additionally, turtle ants also exhibits a high rate of oral-anal trophallaxis [2], which allows young adults (free of symbionts) to consume anal secretions from another adult sister, thus acquiring these core symbionts.

Evidence from previous studies suggests that Burkholderiales, Opitutales, Rhizobiales and Xanthomonadales found in *Cephalotes* samples are involved in upgrading the diet by recycling urea and obtaining nitrogen for the host [4, 33, 35] and also assisting in the formation of the cuticle of the exoskeleton [43]. But these bacteria are not exclusive to *Cephalotes* and some of them have already been identified in other species of ants as well, such as Rhizobiales. This bacterial group has been associated with other herbivorous species such as *Dolichoderus* [64], but also in other species with omnivorous and carnivorous habits such as *Hapergnathos saltator*, *Pheidole*, *Paraponera clavata*, *Daceton armigerum* and army ants [4, 15, 17, 25, 65, 66]. Although the role of this bacterial group is not fully understood, especially in these hosts with a high protein diet, studies suggest that this bacterium may also be related to protein degradation [67].

Another order of bacteria identified in this study that was strongly associated with *Cephalotes* samples in previous works was Opitutales, which was composed almost exclusively of the genus *Cephaloticoccus*, previously found in *Cephalotes* [33, 44, 68]. This bacterium is in high abundance in the midgut of *Cephalotes* samples suggesting that it may assist in the acquisition of nitrogen for the host [33]. Interestingly among the diversity of bacteria associated with turtle ants, our 16S rRNA library was able to recover more aerobic than anaerobic bacteria. The same pattern was also found for another leaf-eating grasshopper insect [69]. Although this needs to be investigated further, this result suggests that the level of oxygenation in these insect guts may be favoring the survival of these bacteria.

Specifically in ants, several studies have already showed that there is structuring of bacterial communities of different species, stage of development within the colony, parts of the body and different tissues, habitats, host phylogeny and even the diet of the host[4, 6, 15, 17, 25–27, 31, 32, 34, 44, 49, 50]. Growing evidence has shown that there is no absolute rule for factors structuring host microbiomes, and that each study system has its particularities and must be considered individually.

In the case of turtle ants, despite having a stable bacterial core community [23, 24, 31, 34, 35] it is possible to find natural variation and structure in these communities when analyzed across different scales. Previous studies have been able to show structuring of *Cephalotes* bacterial communities in different gut chambers [14, 33, 44], and also in different colonies of *Cephalotes varians* [34], and our data also indicate that both belonging to a certain species as well as to a certain biogeographic region contributes to the structuring of the composition of the microbial communities of *Cephalotes*. In addition, species and biogeographical region of the ant host impacted bacterial quantification (copies of 16S rRNA) identified through qPCR. We also found significant differences for the abundance of bacterial communities within the different species of *Cephalotes* analyzed. In addition, we found differences in abundance of bacterial communities in the different biogeographic regions, but the same pattern was not observed for eukaryotic communities.

Interactions between microbial communities and hosts are being increasingly studied, including eukaryotic and bacterial domains [25, 53]. Our data show the synergism or antagonisms between the bacterial and microbial eukaryotic partners within *Cephalotes* hosts. In general, if these interactions of eukaryotic and bacterial communities show mutualism, parasitism, or a tripartite interaction (or more parts) it still deserves to be further explored in future studies. As one of the examples*, Wolbachia* showed positive and negative interactions with several different microbial eukaryotic taxa. This bacteria is one of the most well-known bacteria infecting insects, including social insects [70] whose consequences for host ants have not yet been fully clarified (but see [71, 72]). Thus, the relationship between *Wolbachia* and these other taxa are undescribed and deserves to be explored in depth.

Some symbionts have shown patterns of codiversification with the host, probably facilitated by a high fidelity to vertical transmission, and this is evidenced by the congruence of the host’s phylogenetic tree and the symbiont as already observed in *Buchnera* and aphids [73], *Snodgrassella* and *Gilliamella* bacteria in corbiculate bees [74–76], *Blochmannia* and Camponotini ants [30] and Firmicutes and army ants [15]. Previous studies have found evidence of a correlation between the entire bacterial community and the phylogeny of twenty five species of *Cephalotes*, suggesting that this pattern may reflect codiversification of core bacteria and host or a similar selective environment between related hosts [31].

Approaches that take individual host-associated bacterial groups separately into account should also be explored. For example a study conducted recently with the abdomen/gaster of eleven species of *Cephalotes* were able to find correlation between the bacterial community and the phylogeny of the host [33], a result mainly structured by Opitutales and Burkholderiales. Our study conducted on a broad sampling of *Cephalotes* species shows that for some lineages we failed to find evidence of codiversification with the host turtle ants, and we only found Burkholderiaceae bacterial lineage are codiversifying and being consistently transmitted vertically or may have varying histories of the timing of acquisition or frequent horizontal gene transfer between closely related bacteria. Another suggestion is that some of these bacteria are being picked up from the environment, which may explain the signal from the biogeographic regions structuring the microbial communities in this present study. This same trend was observed in deep-sea anglerfish, despite different species sharing the main symbionts, these are not acquired vertically. In this system, deep-sea anglerfish acquire the symbionts from the environment in every new generation [77].

Horizontal acquisition of beneficial microbes through anal trophallaxis is essential in colonies of bees and termites [78–80]. The social behavior of these insects allows for the constant acquisition and maintenance of microbial partners within individuals of the same colony [19]. Similarly the stability of the core community associated with turtle ants in this study and in several previous studies [4, 31, 34, 35], is likely due to acquisition across generations and between individuals through oral anal trophallaxis [2, 33]. In general, detecting codiversification in horizontally acquired microorganisms is rarer than in vertically acquired microorganisms [81].

## Conclusions

Overall, we have shown that for the turtle ants there is a diverse and evolutionarily stable core bacterial community that is structured by host species and to some extent biogeography of the host. Although we found some signal of microbial interactions between bacteria and microbial eukaryotes the lack of a highly populated microbial 18S rRNA database precluded us from fully exploring these data. We did find codiversification of Burkholderiaceae bacterial lineage with their *Cephalotes* hosts, which leads to interesting questions about what microbial or host factors influence when these partner histories become evolutionarily intertwined.

## Methods

### Collection, DNA extraction and sequencing

For this study we included 192 samples of *Cephalotes*, representing more than 75 different species and covering the entire geographic distribution of the genus. The complete list of specimens and location can be found in Additional file 10. Vouchers of all samples have been deposited in the scientific holdings of entomological collections. After collection, the specimens were preserved in 95% alcohol and stored in the − 20° C freezer.

DNA extraction used DNeasy Blood & Tissue Kits (Qiagen, USA) and DNeasy PowerSoil Kit (Qiagen, USA) with the modification of a beat-beating step and addition of Proteinase K and was performed according to the manufacturer's recommendations. Total DNA was extracted from either from one gaster (abdomen) or one whole worker, or even in the case of material deposited in collections with the non-destructive methodology. Subsequent analyses controlled for these variations so as not to affect the results. Additionally, recommendations by Rubin et al. [82] and Moreau [83] were applied to minimize contamination. In addition, four blank samples were added as negative controls. Our library targeted the amplification of the V4 region of 16S rRNA (primers: 515F “Parada” forward primer, barcoded 5′-AATGATACGGCGACCACCGAGATCTACACGCT XXXXXXXXXXXX TATGGTAATT GT GTGYCAGCMGCCGCGGTAA [84]/806R “Apprill” reverse primer 5’-CAAGCAGAAGACGGCATACGAGAT AGTCAGCCAG CC GGACTACNVGGGTWTCTAAT [85]) and the V9 region of 18S rRNA from microbial eukaryotes (Euk_1391f forward primer 5′-TATCGCCGTT CG GTACACACCGCCCGTC/EukBr reverse primer, barcoded 1510r 5’ CAAGCAGAAGACGGCATACGAGAT XXXXXXXXXXXX AGTCAGTCAG CA TGATCCTTCTGCAGGTTCACCTAC [86, 87]) according to the recommendations of [88], and following the Earth Microbiome Project (EMP) protocol (http://www.earthmicrobiome.org/protocols-and-standards/). For each sample three PCRs were performed containing 12 μl of PCR water (Certified DNA-free), 10 μl of 5 Prime HotMasterMix (1 ×) (5 PRIME, Gaithersburg, USA), 1 μl of forward primer (5 mM concentration, 200 pM final), 1 μl of reverse primer (5 mM concentration, 200 pM final) and 1 μL of template DNA (> 0.20 ng/μl), totaling a final volume of 25 μl per PCR reaction. These reactions were placed in the thermocycler under the following conditions: 94 °C for 3 min, with 35 cycles at 94 °C for 45 s, 50 °C for 60 s, and 72 °C for 90 s, with a final cycle of 10 min at 72 °C. Agarose gel electrophoresis (1%) confirmed the efficiency of the amplification. Quantification by Qubit (Thermo Fisher Scientific) was performed with the High Sensitivity Assay Kit (Life Technologies Corp., Carlsbad, USA). Quantification by qPCR was also performed (see description below). Afterwards, samples were pooled to a total of 100 μL per pool, and purified with QIAquick PCR Purification Kit (Qiagen, USA), following the manufacturer's recommendations. Each pool was diluted to 4 nM and then denatured. Then the pool was further diluted to a final concentration of to 6.75 pM with a 10% PhiX, following Illumina recommendations. Sequencing was performed at Argonne National Laboratory (Lemont, Illinois, USA) with two separate runs (16S and 18S rRNA) through MiSeq Illumina V3 Reagent Kit 600 Cycles (300 × 300) using the custom sequencing primers and procedures described in the supplementary methods in Caporaso et al. [88]. All raw sequence data are publicly available on the NCBI SRA (accession number PRJNA859790 and BioSample SUB11807550).

### Bacterial quantification

Quantification of the abundance of bacteria was performed with real-time qPCR using the universal primers 515f (5′-GTGCCAGCMGCCGCGGTAA) and 806r (5′-GGACTACHVGGGTWTCTAAT) (http://earthmicrobiome.org/emp-standard-protocols/16s/) to target the bacterial 16S rRNA gene using a CFX Connect qPCR machine (Bio-Rad, Hercules, USA), with SYBRAdvanced 2X (Bio-Rad) SYBR green supermix and 2 μL of DNA. All samples were quantified in triplicate. Serial dilutions of plasmids containing inserts of *E. coli* 16S rRNA were performed to establish standard curves [82]. Only reactions that had a R2 from 90 to 110% were considered satisfactory. In order to search for significant differences in bacterial quantification (qPCR) between samples, Analysis of variance (ANOVA) and Wilcoxon tests were applied through Dplyr package [89] in R software [90]. We used ggplot2 [91] to visualize all qPCR results.

### Bioinformatic analysis

Initial analyses were conducted with demultiplexed sequences with Dada2 [92, 93] in Qiime2-2019.10 [94]. Paired-end sequence reads were trimmed for removing primers and maintaining read quality regions. The SILVA 132 QIIME database with 99% similarity [95, 96] was used for taxonomic assignment of ASVs (amplicon sequence variants). Our own classifier was created, and the strings were classified by taxon using the “feature-classifier classify-sklearn” command [97]. In the initial filtering of our data, singleton, mitochondria, and chloroplast taxa were removed. For the 18S rRNA library, Hymenoptera reads were also removed. Four negative controls were used to remove contaminants from the samples through the Decontam package [98] using R with the prevalence method.

The microbial phylogeny was performed using SATé-enabled phylogenetic placement (SEPP) [99, 100]. We used PhyloCore (50%) to determine the main or “core” ASVs present in our library [48], which means that ASVs present in at least 50% of all individuals [46].The core bacteria of the *Cephalotes* species included in this study were visualized through a heat map with the Metacoder package [101] in the environment of R. These ASVs were used in all subsequent analyses. Additionally, we used BugBase [102] to predict aerobic and anaerobic microbial phenotypes for the *Cephalotes* bacterial 16S rRNA library. Alpha and Beta diversity were computed following QIIME2 recommendations [103] and were viewed using the emperor software [104] with 2000 and 1000 reads cutoff for 16S rRNA and 18S rRNA, respectively. To investigate the composition and abundance microbes of *Cephalotes* species permutational multivariate analysis of variance (PERMANOVA) tests were also implemented in QIIME2. To test whether variations in DNA extraction kits or different sample types (gaster, whole worker and non-destructive) impacted our results, PERMANOVA analyses were conducted, and our results show that we only found differences between non-destructive samples. Therefore, these were excluded from our subsequent analyses, with 151 samples remaining.

Subsequent analyses tested the influence of both: (I) host species identity and (II) geographical distribution of the host turtle ants on both microbial communities. For the latter, we followed the recommendation of Price et al. [105], and divided the Neotropical region into eight biogeographic regions based on Morrone’s [106] classification: Amazonian, Antillean, Chacoan, Mesoamerican, Mexican transition zone, Nearctic, Northwestern South American and Paraná. To better visualize the influence of these factors on the bacterial communities in the present study, we use PCoA plots showing results of composition and abundance that considers the influence of phylogenetic signal (weighted and unweighted unifrac) and NMDS analysis (Bray Curtis) as well. We also investigated the distribution of the main/core ASVs associated with turtle ants for the different species of the host as well as the different biogeographic regions. All of these analyses were conducted using phyloseq [107] and ggplot2 (Wickham 2009) packages in the R environment.

### Correlation of the 16S rRNA 18Sr RNA libraries

To test the potential for correlation of the 16S rRNA and 18S rRNA libraries obtained in the present study we performed a Regularized Generalized Canonical Correlation Analysis (rGCCA) through the mixOmics package [108, 109] in the R environment. The visualization of these correlations was implemented in Cytoscape 3.5.1 [110] and the heatmap was implemented in R software.

### Testing coevolution between core bacteria and turtle ant hosts

Each microbial lineage has an independent and complex evolutionary history with the host, therefore two methods were applied to test for coevolutionary signal between the core gut bacteria (50%) and the host’s evolutionary history: Mantel test and PACo (Procrustean Approach to Cophylogeny) analyses [111, 112]. For this, the host *Cephalotes* phylogenomic tree of Price et al. [45] was used in the following analyses. First, the 10 main strains of bacteria recovered were analyzed separately because we know that each bacterial lineage may have a different evolutionary history. For the Mantel test, the ASV tables of each of the 10 bacterial strains were transformed into distance (Bray Curtis metric) as well as the host phylogenetic tree, and the correlation of the two matrices was calculated using the Mantel test (999 permutations) using the Vegan package [113] in R. The PACo analysis explicitly tests the dependence of the phylogeny of the symbiont, in the case of the present study of host-associated bacteria, against the phylogeny of the host turtle ants [111, 112]. For this, the program needs three inputs: two phylogenetic trees with branch lengths one each from the host and bacteria, and a binary matrix that encodes symbiont-host associations.

For a better visualization of host-bacterial associations, tanglegrams were plotted with the Phytools package [114] in the R environment using the cophylo function. Hence, phylogenetic trees were transformed into matrices of pairwise patristic distance, then into matrices of principal coordinates, and these are subjected to the analysis of Procrustes with global goodness-of-fit statistic with significance tested by random permutations (10,000 permutations).

## Supplementary Information

**Additional file 1**. Sample type differences between samples of* Cephalotes* used in this study. Note that there is no difference between DNA extract kits and the abdomen/gaster and the whole worker

**Additional file 2**. Aerobic and anaerobic phenotype bacteria identify in turtle ants. BugBase results for 16S rRNA in* Cephalotes* in the present study.

**Additional file 3**. Bacterial quantification (qPCR) through the 16S rRNA gene (515F/806R) associated* Cephalotes* samples. Note that belonging to a species and a biogeographic region impact the results of the quantity of bacteria (number of copies of 16S rRNA) in the host.

**Additional file 4**. Species and biogeographic regions that impact qPCR results. Group 1 consists of the average of the entire dataset to be compared with group 2. Those that presented significant results are highlighted in red.

**Additional file 5**. Alpha diversity found in samples of* Cephalotes* measured by Shannon and Simpson indices.** A** High alpha diversity found in bacterial communities associated with turtle ants.** B** Alpha diversity in eukaryotic microbials associated with turtle ants is lower compared to the bacterial community. Note included in these visualizations alpha diversity can be compared for different species as well as by different biogeographic regions.

**Additional file 6**. Kruskal–Wallis pairwise tests of combinations of species of* Cephalotes* that showed significant results.

**Additional file 7**. Bar plots illustrating the abundance of the most common bacterial orders associated with turtle ants.** A** Main orders of ASVs are grouped according to different host species of* Cephalotes*.** B** Main orders of ASVs grouped according to different biogeographic regions of* Cephalotes* distribution

**Additional file 8**. Bar plots illustrating the abundance of most common Eukaryotic orders associated with turtle ants.** A** Main orders of ASVs grouped according to different host species of* Cephalotes*.** B** Main orders of ASVs grouped according to different biogeographic regions of* Cephalotes* distribution.

**Additional file 9**. Non-metric multidimensional scaling (NMDS) (Bray-Curtis) ordination of bacterial communities of* Cephalotes* samples colored according to different species and biogeographic regions with 95% confidence interval

**Additional file 10**. List of specimens included in this study.

## Data Availability

All raw sequence data are publicly available NCBI SRA accession number PRJNA859790 and BioSample SUB11807550.

## References

[1] Colwell RK, Coddington JA (1994). Estimating terrestrial biodiversity through extrapolation. Philos Trans R Soc Lond B Biol Sci.

[2] Hölldobler B, Wilson EO (1990). The ants.

[3] Moreau CS, Bell CD, Vila R, Archibald SB, Pierce NE (2006). Phylogeny of the ants: diversification in the age of angiosperms. Science.

[4] Russell JA, Moreau CS, Goldman-Huertas B, Fujiwara M, Lohman DJ, Pierce NE (2009). Bacterial gut symbionts are tightly linked with the evolution of herbivory in ants. Proc Natl Acad Sci U.S.A..

[5] Davidson DW, Cook SC, Snelling RR, Chua TH (2003). Explaining the abundance of ants in lowland tropical rainforest canopies. Science.

[6] Sanders JG, Łukasik P, Frederickson ME, Russell JA, Koga R, Knight R (2017). Dramatic differences in gut bacterial densities correlate with diet and habitat in rainforest ants. Integr Comp Biol.

[7] Schultz TR, Brady SG (2008). Major evolutionary transitions in ant agriculture. Proc Nat Acad Sci..

[8] Pringle EG, Moreau CS (2017). Community analysis of microbial sharing and specialization in a Costa Rican ant–plant–hemipteran symbiosis. Proc R Soc B Biol Sci.

[9] Nixon G (1951). The association of ants with aphids and coccids.

[10] Del-Claro K, Oliveira PS (1993). Ant-Homoptera interaction: Do alternative sugar sources distract tending ants?. Oikos.

[11] Russell JA, Funaro CF, Giraldo YM, Goldman-Huertas B, Suh D, Kronauer DJC (2012). A veritable menagerie of heritable bacteria from ants, butterflies, and beyond: broad molecular surveys and a systematic review. PLoS ONE.

[12] Buchner P (1965). Endosymbiosis of animals with plant microorganisms.

[13] Feldhaar H, Straka J, Krischke M, Berthold K, Stoll S, Mueller M (2007). Nutritional upgrading for omnivorous carpenter ants by the endosymbiont Blochmannia. BMC Biol.

[14] Kautz S, Rubin BER, Russell JA, Moreau CS (2013). Surveying the microbiome of ants: comparing 454 pyrosequencing with traditional methods to uncover bacterial diversity. Appl Environ Microbiol.

[15] Łukasik P, Newton JA, Sanders JG, Hu Y, Moreau CS, Kronauer DJC (2017). The structured diversity of specialized gut symbionts of the New World army ants. Mol Ecol.

[16] Moreau CS, Rubin BER (2017). Diversity and persistence of the gut microbiome of the giant neotropical bullet ant. Integr Comp Biol.

[17] Martins C, Moreau CS (2020). Influence of host phylogeny, geographical location and seed harvesting diet on the bacterial community of globally distributed Pheidole ants. PeerJ.

[18] Moreau CS (2020). Symbioses among ants and microbes. Curr Opin Insect Sci.

[19] Salem H, Kaltenpoth M, Florez L, Gerardo N (2015). An out-of-body experience: the extracellular dimension for the transmission of mutualistic bacteria in insects. Proc R Soc B.

[20] Van Borm S, Wenseleers T, Billen J, Boomsma JJ (2008). *Wolbachia* in leafcutter ants: a widespread symbiont that may induce male killing or incompatible matings. J Evol Biol.

[21] Cook SC, Davidson DW (2006). Nutritional and functional biology of exudate-feeding ants. Entomol Exp Appl.

[22] Stoll S, Gadau J, Gross R, Feldhaar H (2007). Bacterial microbiota associated with ants of the genus *Tetraponera*. Biol J Linn Soc.

[23] Bution M, Caetano F (2008). Ileum of the *Cephalotes* ants: a specialized structure to harbor symbionts microorganisms. Micron.

[24] Bution ML, Caetano FH (2010). The midgut of *Cephalotes* ants (Formicidae: Myrmicinae): ultrastructure of the epithelium and symbiotic bacteria. Micron.

[25] Ramalho M, Duplais C, Orivel J, Gibson J, Dejan A, Suarez A (2020). Development but not diet alters microbial communities in the Neotropical arboreal trap jaw ant *Daceton armigerum*: an exploratory study. Sci Rep.

[26] Vieira AS, Ramalho MO, Martins C, Martins VG, Bueno OC (2017). Microbial communities in different tissues of *Atta sexdens rubropilosa* leaf-cutting ants. Curr Microbiol.

[27] Ramalho M, Martins C, Morini M, Bueno O (2020). What can the bacterial community of *Atta sexdens* (Linnaeus, 1758) tell us about the habitats in which this ant species evolves?. Insects.

[28] Kautz S, Rubin BER, Moreau CS (2013). Bacterial infections across the ants: frequency and prevalence of *Wolbachia*, *Spiroplasma*, and *Asaia*. Psyche A J Entomol.

[29] Song SJ, Sanders JG, Delsuc F, Metcalf J, Amato K, Taylor MW (2020). Comparative analyses of vertebrate gut microbiomes reveal convergence between birds and bats. MBio.

[30] Wernegreen JJ, Kauppinen SN, Brady SG, Ward PS (2009). One nutritional symbiosis begat another: phylogenetic evidence that the ant tribe Camponotini acquired *Blochmannia* by tending sap-feeding insects. BMC Evol Biol.

[31] Sanders JG, Powell S, Kronauer DJC, Vasconcelos HL, Frederickson ME, Pierce NE (2014). Stability and phylogenetic correlation in gut microbiota: lessons from ants and apes. Mol Ecol.

[32] Ramalho MO, Bueno OC, Moreau CS (2017). Microbial composition of spiny ants (Hymenoptera: Formicidae: *Polyrhachis*) across their geographic range. BMC Evol Biol.

[33] Flynn PJ, D’Amelio CL, Sanders JG, Russell JA, Moreau CS (2021). Localization of bacterial communities within gut compartments across *Cephalotes* turtle ants. Appl Environ Microbiol.

[34] Hu Y, Łukasik P, Moreau CS, Russell JA (2014). Correlates of gut community composition across an ant species (*Cephalotes varians*) elucidate causes and consequences of symbiotic variability. Mol Ecol.

[35] Hu Y, Sanders JG, Łukasik P, D’Amelio CL, Millar JS, Vann DR (2018). Herbivorous turtle ants obtain essential nutrients from a conserved nitrogen-recycling gut microbiome. Nat Commun.

[36] De Andrade M, Urbani C. Diversity and adaptation in the ant genus *Cephalotes*, past and present. In: tuttgarter Beitraege Zur Naturkunde. Serie B: Geologie Und Palaeontologie. 1999.

[37] Byk J, Del-Claro K (2010). Nectar-and pollen-gathering *Cephalotes* ants provide no protection against herbivory: a new manipulative experiment to test ant protective capabilities. Acta Ethol.

[38] Gordon DM (2012). The dynamics of foraging trails in the tropical arboreal ant *Cephalotes goniodontus*. PLoS ONE.

[39] Weber NA (1957). The nest of an anomalous colony of the arboreal ant *Cephalotes* atratus. Psyche (New York).

[40] Adams ES (1990). Interaction between the ants Zacryptocerus maculatus and Azteca trigona: interspecific parasitization of information. Biotropica.

[41] Jaffe K, Caetano FH, Sánchez P, Hernández JV, Caraballo L, Vitelli-Flores J (2001). Sensitivity of ant (*Cephalotes*) colonies and individuals to antibiotics implies feeding symbiosis with gut microorganisms. Can J Zool.

[42] Powell S (2008). Ecological specialization and the evolution of a specialized caste in *Cephalotes* ants. Funct Ecol.

[43] Duplais C, Sarou-Kanian V, Massiot D, Hassan A, Perrone B, Estevez Y (2021). Gut bacteria are essential for normal cuticle development in herbivorous turtle ants. Nat Commun.

[44] Lanan MC, Augusto P, Rodrigues P, Agellon A, Jansma P, Wheeler DE. A bacterial filter protects and structures the gut microbiome of an insect. ISME J. 2015;12. 10.1038/ismej.2015.264 PMC5029173 26872040

[45] Price SL, Blanchard BD, Powell S, Blaimer BB, Moreau CS (2022). Phylogenomics and fossil data inform the systematics and geographic range evolution of a diverse Neotropical ant lineage. Insect Syst Divers.

[46] Astudillo-García C, Bell JJ, Webster NS, Glasl B, Jompa J, Montoya JM (2017). Evaluating the core microbiota in complex communities: a systematic investigation. Environ Microbiol.

[47] Shade A, Handelsman J (2012). Beyond the Venn diagram: the hunt for a core microbiome. Environ Microbiol.

[48] Ren T, Wu M (2016). PhyloCore: a phylogenetic approach to identifying core taxa in microbial communities. Gene.

[49] Ramalho MO, Bueno OC, Moreau CS (2017). Species-specific signatures of the microbiome from *Camponotus* and *Colobopsis* ants across developmental stages. PLoS ONE.

[50] Ramalho MO, Moreau CS, Bueno OC (2019). The potential role of environment in structuring the microbiota of *Camponotus* across parts of the body. Adv Entomol.

[51] De Oliveira TB, Ferro M, Bacci M, De Souza DJ, Fontana R, Delabie JHC (2016). Bacterial communities in the midgut of Ponerine ants (Hymenoptera: Formicidae: Ponerinae). Scopus.

[52] Harmon LJ, Glor RE (2010). Poor statistical performance of the mantel test in phylogenetic comparative analyses. Evolution (N Y).

[53] Schuelke T, Pereira TJ, Hardy SM, Bik HM (2018). Nematode-associated microbial taxa do not correlate with host phylogeny, geographic region or feeding morphology in marine sediment habitats. Mol Ecol.

[54] Groussin M, Mazel F, Sanders JG, Smillie CS, Lavergne S, Thuiller W (2017). Unraveling the processes shaping mammalian gut microbiomes over evolutionary time. Nat Commun.

[55] Douglas AE (2015). Multiorganismal insects: diversity and function of resident microorganisms. Annu Rev Entomol.

[56] McFall-Ngai M, Hadfield MG, Bosch TCG, Carey HV, Domazet-Lošo T, Douglas AE (2013). Animals in a bacterial world, a new imperative for the life sciences. Proc Natl Acad Sci U.S.A..

[57] Moran NA, McCutcheon JP, Nakabachi A (2008). Genomics and evolution of heritable bacterial symbionts. Annu Rev Genet.

[58] Dheilly NM, Bolnick D, Bordenstein S, Brindley PJ, Figuères C, Holmes EC (2017). Parasite microbiome project: systematic investigation of microbiome dynamics within and across parasite-host interactions. MSystems.

[59] Nunes-Alves C (2015). Commensal bacterium prevents wasting. Nat Rev Microbiol.

[60] Parfrey LW, Walters WA, Knight R (2011). Microbial eukaryotes in the human microbiome: ecology, evolution, and future directions. Front Microbiol.

[61] Rubin BE, Kautz S, Wray BD, Moreau CS (2019). Dietary specialization in mutualistic acacia-ants affects relative abundance but not identity of host-associated bacteria. Mol Ecol..

[62] Kwong WK, Medina LA, Koch H, Sing KW, Soh EJY, Ascher JS (2017). Dynamic microbiome evolution in social bees. Sci Adv.

[63] Brown BP, Wernegreen JJ (2016). Deep divergence and rapid evolutionary rates in gut-associated Acetobacteraceae of ants. BMC Microbiol.

[64] Bisch G, Neuvonen M-M, Pierce NE, Russell JA, Koga R, Sanders JG (2018). Genome evolution of Bartonellaceae symbionts of ants at the opposite ends of the trophic scale. Genome Biol Evol.

[65] Larson HK, Goffredi SK, Parra EL, Vargas O, Pinto-Tomas AA, McGlynn TP (2014). Distribution and dietary regulation of an associated facultative Rhizobiales-related bacterium in the omnivorous giant tropical ant, *Paraponera clavata*. Naturwissenschaften.

[66] Neuvonen M-M, Tamarit D, Näslund K, Liebig J, Feldhaar H, Moran NA (2016). The genome of Rhizobiales bacteria in predatory ants reveals urease gene functions but no genes for nitrogen fixation. Sci Rep.

[67] Dussutour A, Simpson SJ (2012). Ant workers die young and colonies collapse when fed a high-protein diet. Proc R Soc B Biol Sci.

[68] Lin JY, Russell JA, Sanders JG, Wertz JT (2016). Cephaloticoccus gen. Nov., a new genus of ‘Verrucomicrobia’ containing two novel species isolated from *Cephalotes* ant guts. Int J Syst Evol Microbiol.

[69] Zhu L, Zhang Y, Cui X, Zhu Y, Dai Q, Chen H (2021). Host bias in diet-source microbiome transmission in wild cohabitating herbivores: new knowledge for the evolution of herbivory and plant defense. Microbiol Spectr.

[70] Ramalho MDO, Kim Z, Wang S, Moreau CS (2021). *Wolbachia* across social insects: patterns and Implications. Ann Entomol Soc Am.

[71] Singh R, Linksvayer TA (2020). *Wolbachia*-infected ant colonies have increased reproductive investment and an accelerated life cycle. J Exp Biol.

[72] Tseng SP, Wetterer JK, Suarez AV, Lee CY, Yoshimura T, Shoemaker DW (2019). Genetic diversity and *Wolbachia* infection patterns in a globally distributed invasive ant. Front Genet.

[73] Douglas AE (1998). Nutritional interactions in insect-microbial symbioses: aphids and their symbiotic bacteria *Buchnera*. Annu Rev Entomol.

[74] Engel P, Martinson VG, Moran NA (2012). Functional diversity within the simple gut microbiota of the honey bee. Proc Natl Acad Sci U.S.A..

[75] Kwong WK, Moran NA (2015). Evolution of host specialization in gut microbes: the bee gut as a model. Gut Microbes.

[76] Koch H, Abrol DP, Li J, Schmid-Hempel P (2013). Diversity and evolutionary patterns of bacterial gut associates of corbiculate bees. Mol Ecol.

[77] Baker LJ, Freed LL, Easson CG, Lopez JV, Fenolio D, Sutton TT (2019). Diverse deep-sea anglerfishes share a genetically reduced luminous symbiont that is acquired from the environment. Elife.

[78] Koch H, Schmid-Hempel P (2011). Socially transmitted gut microbiota protect bumble bees against an intestinal parasite. Proc Natl Acad Sci U.S.A..

[79] Kitade O (2004). Comparison of symbiotic flagellate faunae between termites and a wood-feeding cockroach of the genus *Cryptocercus*. Microbes Environ.

[80] Köhler T, Dietrich C, Scheffrahn RH, Brune A (2012). High-resolution analysis of gut environment and bacterial microbiota reveals functional compartmentation of the gut in wood-feeding higher termites (*Nasutitermes* spp.). Appl Environ Microbiol.

[81] O’brien PA, Webster NS, Miller DJ, Bourne DG (2019). Host-microbe coevolution: applying evidence from model systems to complex marine invertebrate holobionts. MBio.

[82] Rubin BER, Sanders JG, Hampton-Marcell J, Owens SM, Gilbert JA, Moreau CS (2014). DNA extraction protocols cause differences in 16S rRNA amplicon sequencing efficiency but not in community profile composition or structure. Microbiologyopen.

[83] Moreau CS (2014). A practical guide to DNA extraction, PCR, and gene-based DNA sequencing in insects. Halteres.

[84] Parada AE, Needham DM, Fuhrman JA (2016). Every base matters: assessing small subunit rRNA primers for marine microbiomes with mock communities, time series and global field samples. Environ Microbiol.

[85] Apprill A, Mcnally S, Parsons R, Weber L (2015). Minor revision to V4 region SSU rRNA 806R gene primer greatly increases detection of SAR11 bacterioplankton. Aquat Microb Ecol.

[86] Amaral-Zettler LA, McCliment EA, Ducklow HW, Huse SM (2009). A method for studying protistan diversity using massively parallel sequencing of V9 hypervariable regions of small-subunit ribosomal RNA genes. PLoS ONE.

[87] Stoeck T, Bass D, Nebel M, Christen R, Jones MDM, Breiner HW (2010). Multiple marker parallel tag environmental DNA sequencing reveals a highly complex eukaryotic community in marine anoxic water. Mol Ecol.

[88] Caporaso JG, Lauber CL, Walters WA, Berg-Lyons D, Huntley J, Fierer N (2012). Ultra-high-throughput microbial community analysis on the Illumina HiSeq and MiSeq platforms. ISME J.

[89] Wickham H, François R, Henry L, Müller K. Dplyr: a grammar of data manipulation. R package version 0.4. 3. 2015. 2018.

[90] R Development Core Team (2019) R: A Language and Environment for Statistical Computing. Available from http://www.R-project.org/. 2019.

[91] Wickham H (2009). ggplot2: elegant graphics for data analysis.

[92] Callahan BJ, McMurdie PJ, Rosen MJ, Han AW, Johnson AJA, Holmes SP (2016). DADA2: high-resolution sample inference from Illumina amplicon data. Nat Methods.

[93] McDonald D, Clemente JC, Kuczynski J, Rideout JR, Stombaugh J, Wendel D (2012). The biological observation matrix (BIOM) format or: how i learned to stop worrying and love the ome-ome. Gigascience.

[94] Bolyen E, Rideout JR, Dillon MR, Bokulich NA, Abnet CC, Al-Ghalith GA (2019). Reproducible, interactive, scalable and extensible microbiome data science using QIIME 2. Nat Biotechnol.

[95] Quast C, Pruesse E, Yilmaz P, Gerken J, Schweer T, Yarza P (2013). The SILVA ribosomal RNA gene database project: improved data processing and web-based tools. Nucleic Acids Res.

[96] Yilmaz P, Parfrey LW, Yarza P, Gerken J, Pruesse E, Quast C (2014). The SILVA and “all-species living tree project (LTP)” taxonomic frameworks. Nucleic Acids Res.

[97] Bokulich NA, Kaehler BD, Rideout JR, Dillon M, Bolyen E, Knight R (2018). Optimizing taxonomic classification of marker-gene amplicon sequences with QIIME 2’s q2-feature-classifier plugin. Microbiome.

[98] Davis NM, Proctor D, Holmes SP, Relman DA, Callahan BJ (2018). Simple statistical identification and removal of contaminant sequences in marker-gene and metagenomics data. Microbiome.

[99] Janssen S, McDonald D, Gonzalez A, Navas-Molina JA, Jiang L, Xu ZZ (2018). Phylogenetic placement of exact amplicon sequences improves associations with clinical information. MSystems.

[100] Mirarab S, Nguyen N, Warnow T. SEPP: SATé-enabled phylogenetic placement. In: Pacific Symposium on Biocomputing. 2012. p. 247–58. 10.1142/9789814366496_0024 22174280

[101] Foster ZSL, Sharpton TJ, Grünwald NJ (2017). Metacoder: an R package for visualization and manipulation of community taxonomic diversity data. PLOS Comput Biol.

[102] Ward T, Larson J, Meulemans J, Hillmann B, Lynch J, Sidiropoulos D, et al. BugBase predicts organism-level microbiome phenotypes. bioRxiv. 2017:133462.

[103] Anderson MJ (2001). A new method for non-parametric multivariate analysis of variance. Austral Ecol.

[104] Vázquez-Baeza Y, Pirrung M, Gonzalez A, Knight R (2013). EMPeror: a tool for visualizing high-throughput microbial community data. Gigascience.

[105] Price SL, Powell S, Kronauer DJC, Tran LAP, Pierce NE, Wayne RK (2014). Renewed diversification is associated with new ecological opportunity in the Neotropical turtle ants. J Evol Biol.

[106] Morrone JJ (2006). Biogeographic areas and transition zones of Latin America and the Caribbean Islands based on panbiogeographic and cladistic analyses of the entomofauna. Annu Rev Entomol.

[107] McMurdie PJ, Holmes S (2013). phyloseq: an R package for reproducible interactive analysis and graphics of microbiome census data. PLoS ONE.

[108] Tenenhaus A, Tenenhaus M (2011). Regularized generalized canonical correlation analysis. Psychometrika.

[109] Rohart F, Gautier B, Singh A, Lê Cao K-A (2017). mixOmics: an R package for ‘omics feature selection and multiple data integration. PLOS Comput Biol.

[110] Shannon P, Markiel A, Ozier O, Baliga NS, Wang JT, Ramage D (2003). Cytoscape: a software environment for integrated models of biomolecular interaction networks. Genome Res.

[111] Balbuena JA, Míguez-Lozano R, Blasco-Costa I (2013). PACo: a novel procrustes application to cophylogenetic analysis. PLoS ONE.

[112] Hutchinson MC, Cagua EF, Balbuena JA, Stouffer DB, Poisot T (2017). paco: implementing procrustean approach to cophylogeny in R. Methods Ecol Evol.

[113] Oksanen J, Kindt R, Legendre P, O’Hara B (2007). The vegan package. Community Ecol.

[114] Revell LJ (2012). phytools: an R package for phylogenetic comparative biology (and other things). Methods Ecol Evol.

