# Supplementary figures and images for: Untangling the complex interactions between turtle ants and their microbial partners
Source: Anim Microbiome. 2023 Jan 3;5:1. doi: 10.1186/s42523-022-00223-7 (PMC9809061; doi:10.1186/s42523-022-00223-7)

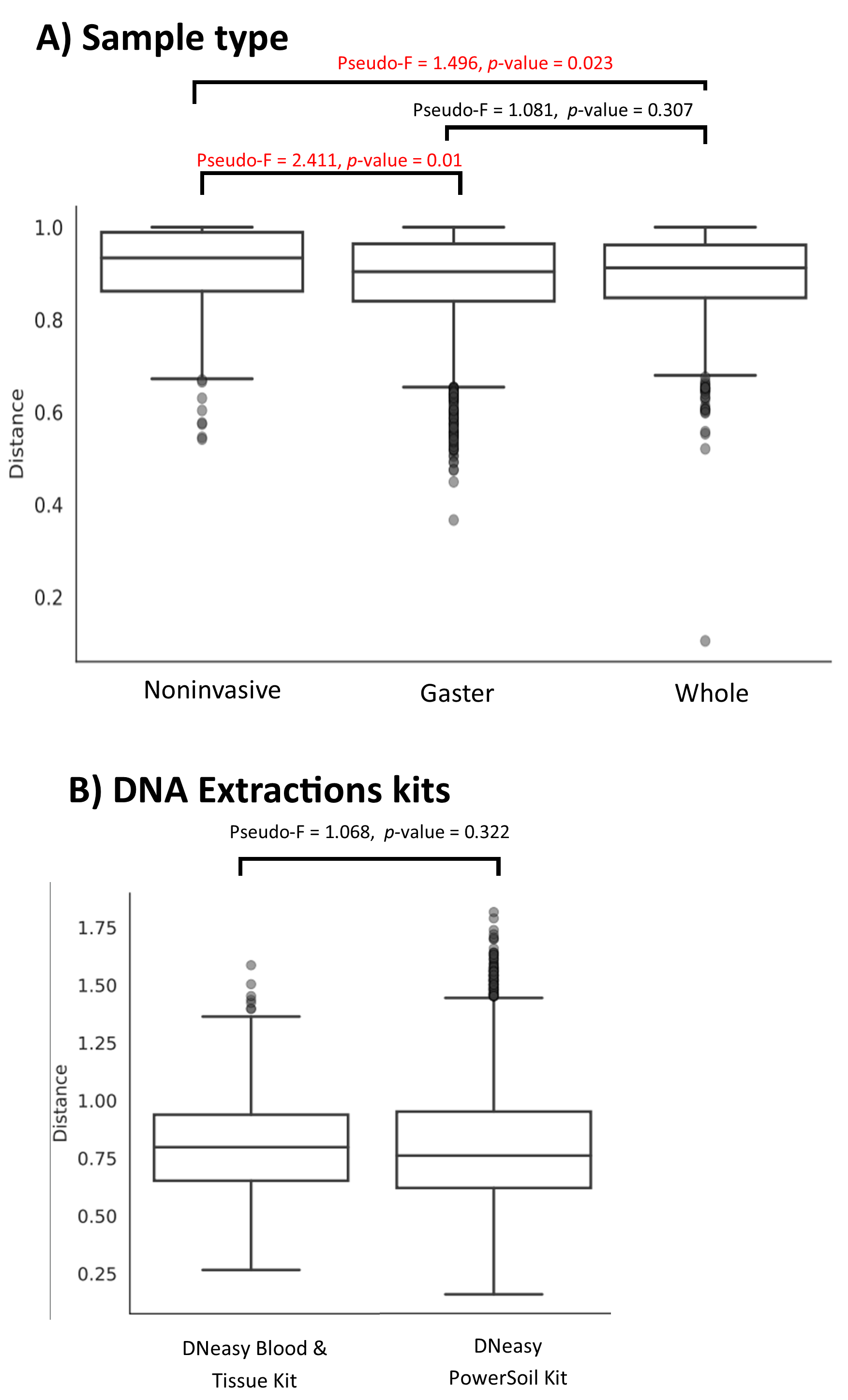

Supplement: Supplementary file 1 — Additional file 1. Sample type differences between samples of Cephalotes used in this study. Note that there is no difference between DNA extract kits and the abdomen/gaster and the whole worker [file 42523_2022_223_MOESM1_ESM.tif]

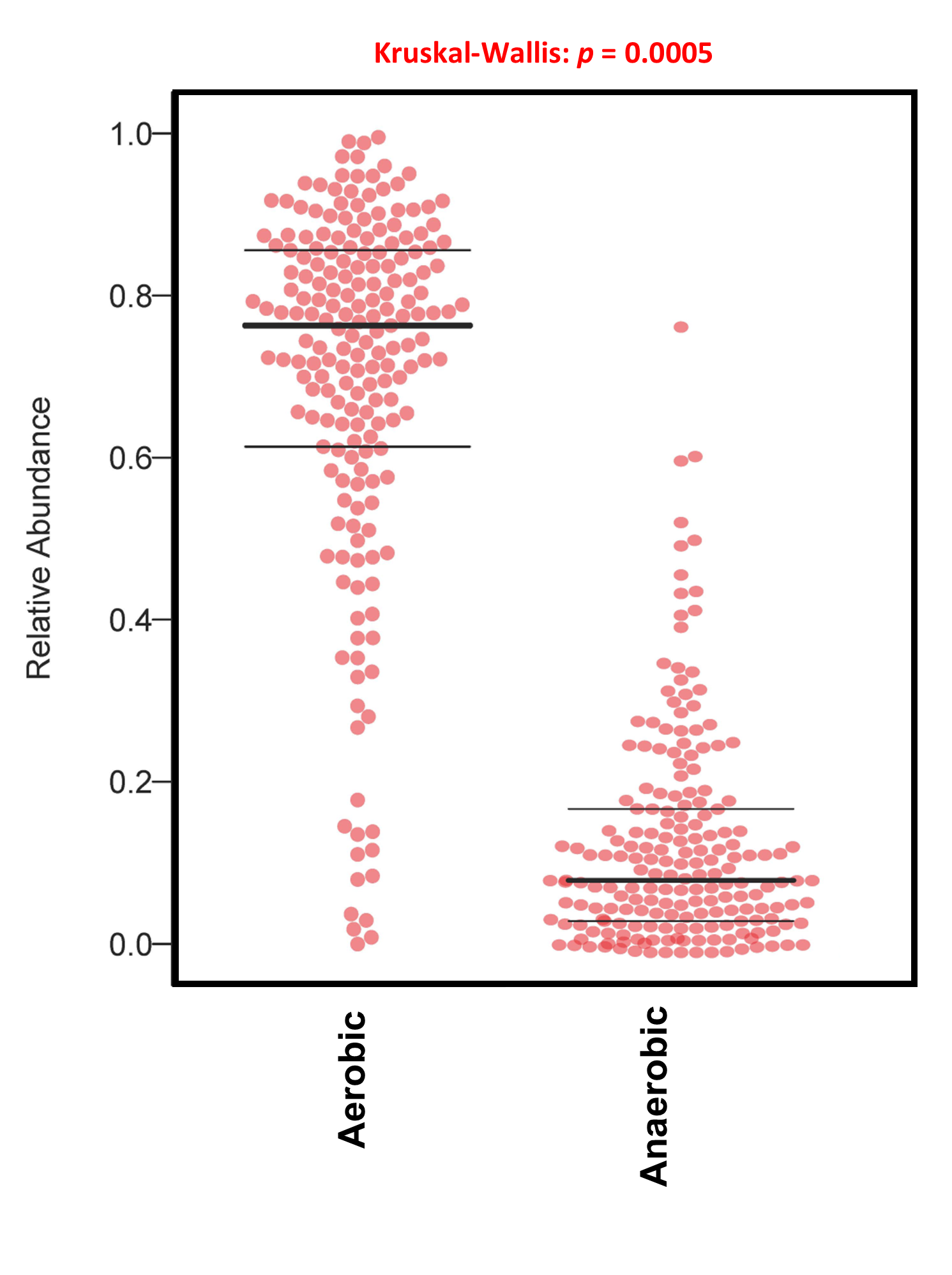

Supplement: Supplementary file 2 — Additional file 2. Aerobic and anaerobic phenotype bacteria identify in turtle ants. BugBase results for 16S rRNA in Cephalotes in the present study. [file 42523_2022_223_MOESM2_ESM.tif]

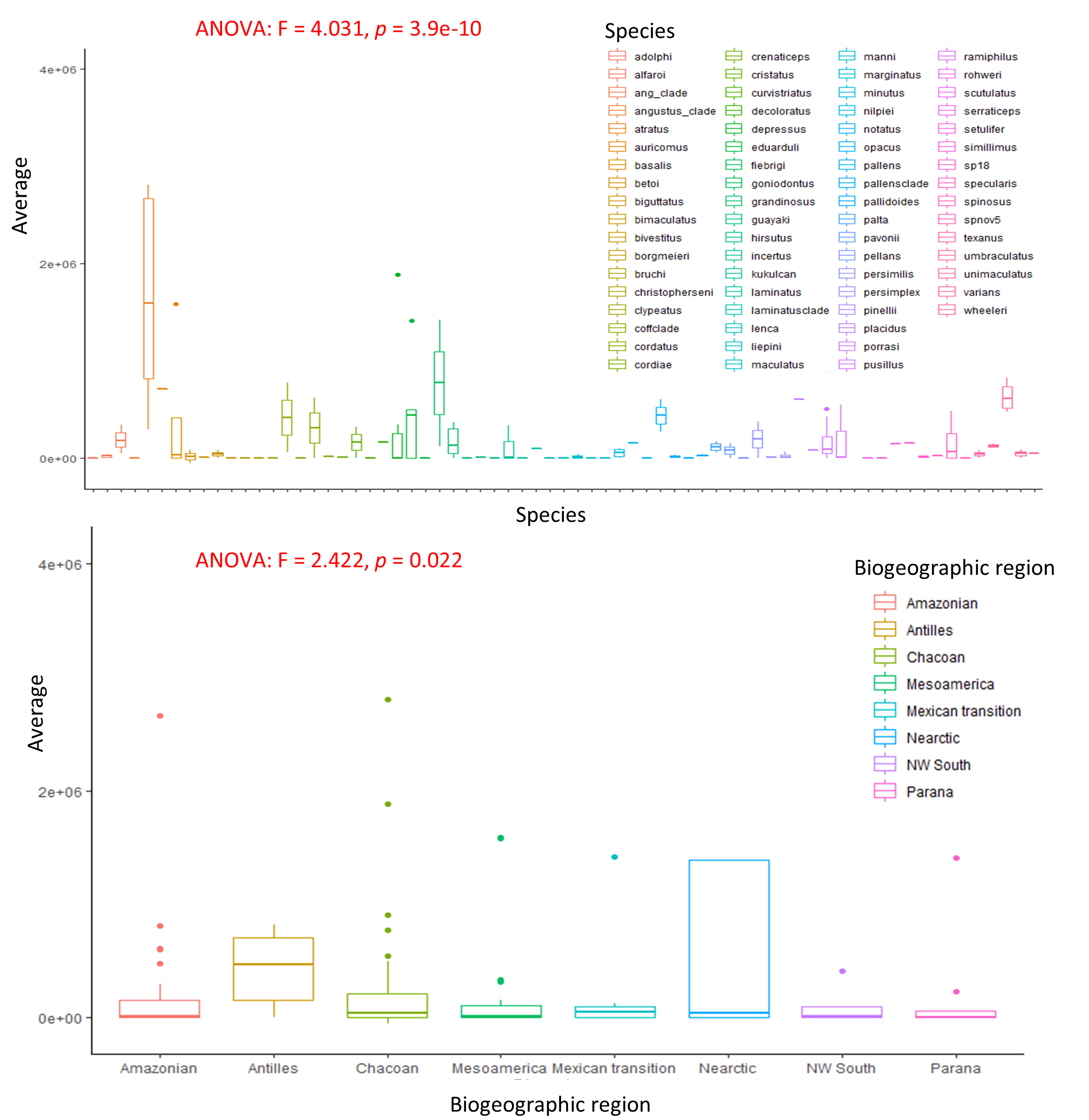

Supplement: Supplementary file 3 — Additional file 3. Bacterial quantification (qPCR) through the 16S rRNA gene (515F/806R) associated Cephalotes samples. Note that belonging to a species and a biogeographic region impact the results of the quantity of bacteria (number of copies of 16S rRNA) in the host. [file 42523_2022_223_MOESM3_ESM.tif]

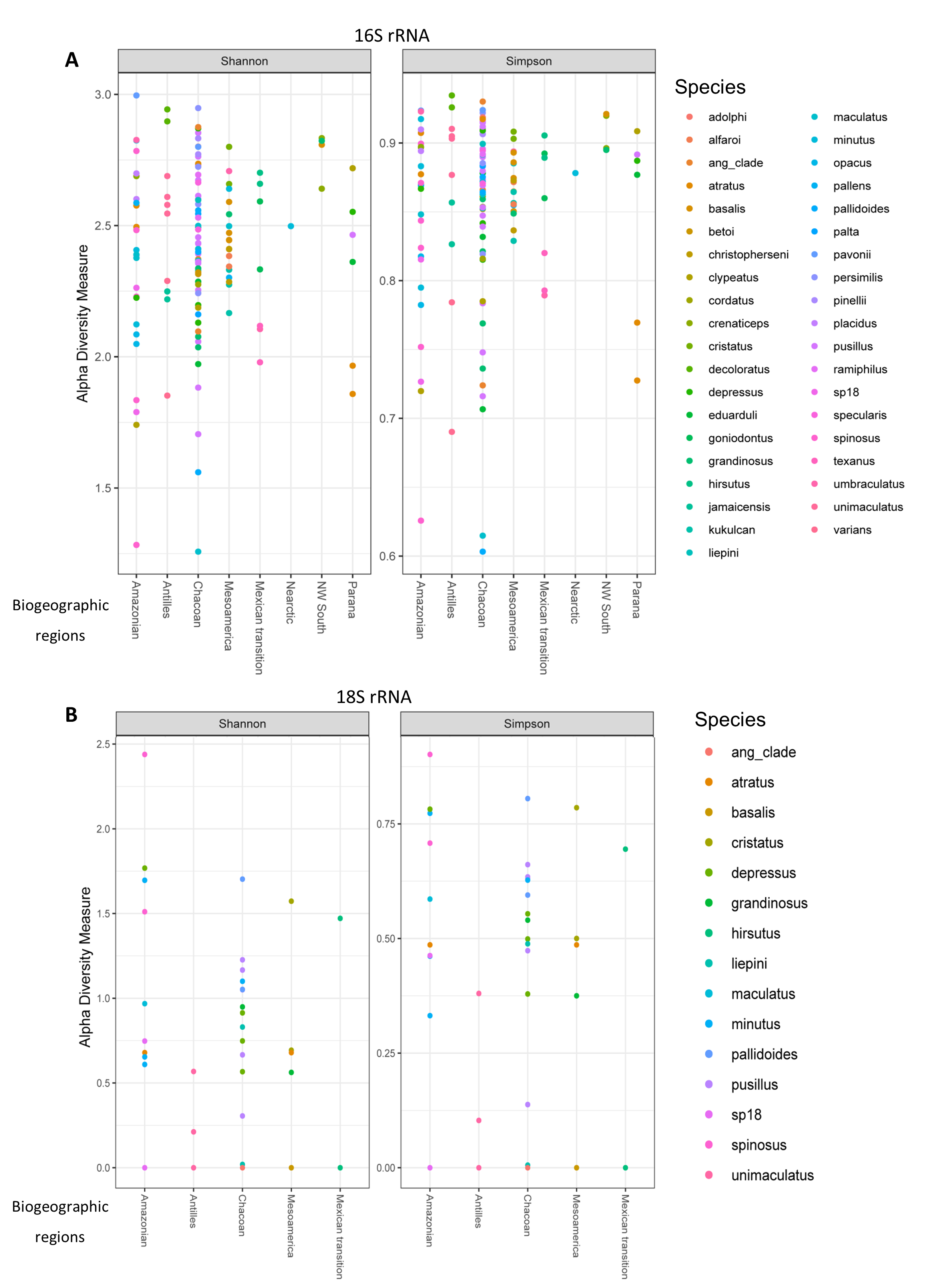

Supplement: Supplementary file 5 — Additional file 5. Alpha diversity found in samples of Cephalotes measured by Shannon and Simpson indices. A High alpha diversity found in bacterial communities associated with turtle ants. B Alpha diversity in eukaryotic microbials associated with turtle ants is lower compared to the bacterial community. Note included in these visualizations alpha diversity can be compared for different species as well as by different biogeographic regions. [file 42523_2022_223_MOESM5_ESM.tif]

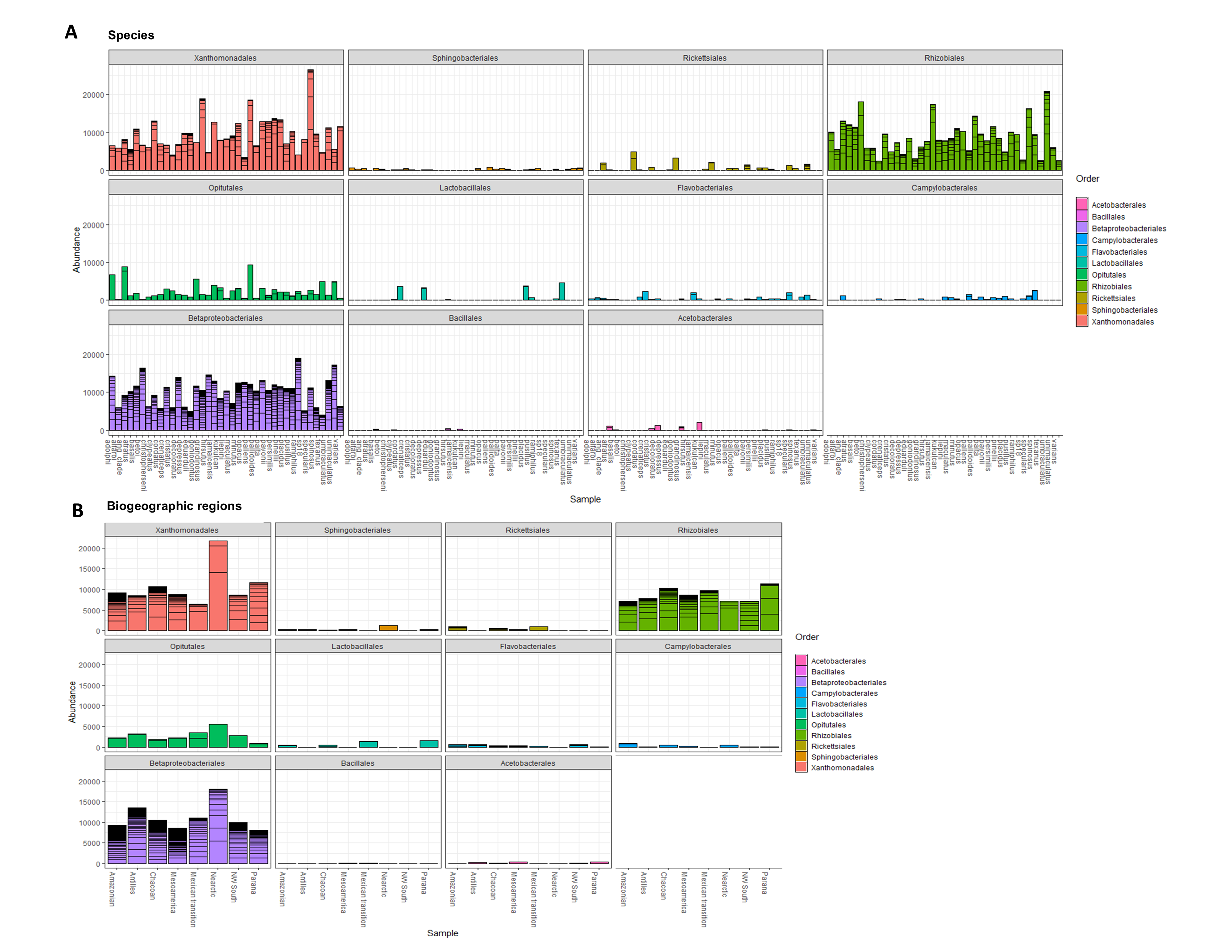

Supplement: Supplementary file 7 — Additional file 7. Bar plots illustrating the abundance of the most common bacterial orders associated with turtle ants. A Main orders of ASVs are grouped according to different host species of Cephalotes. B Main orders of ASVs grouped according to different biogeographic regions of Cephalotes distribution [file 42523_2022_223_MOESM7_ESM.tif]

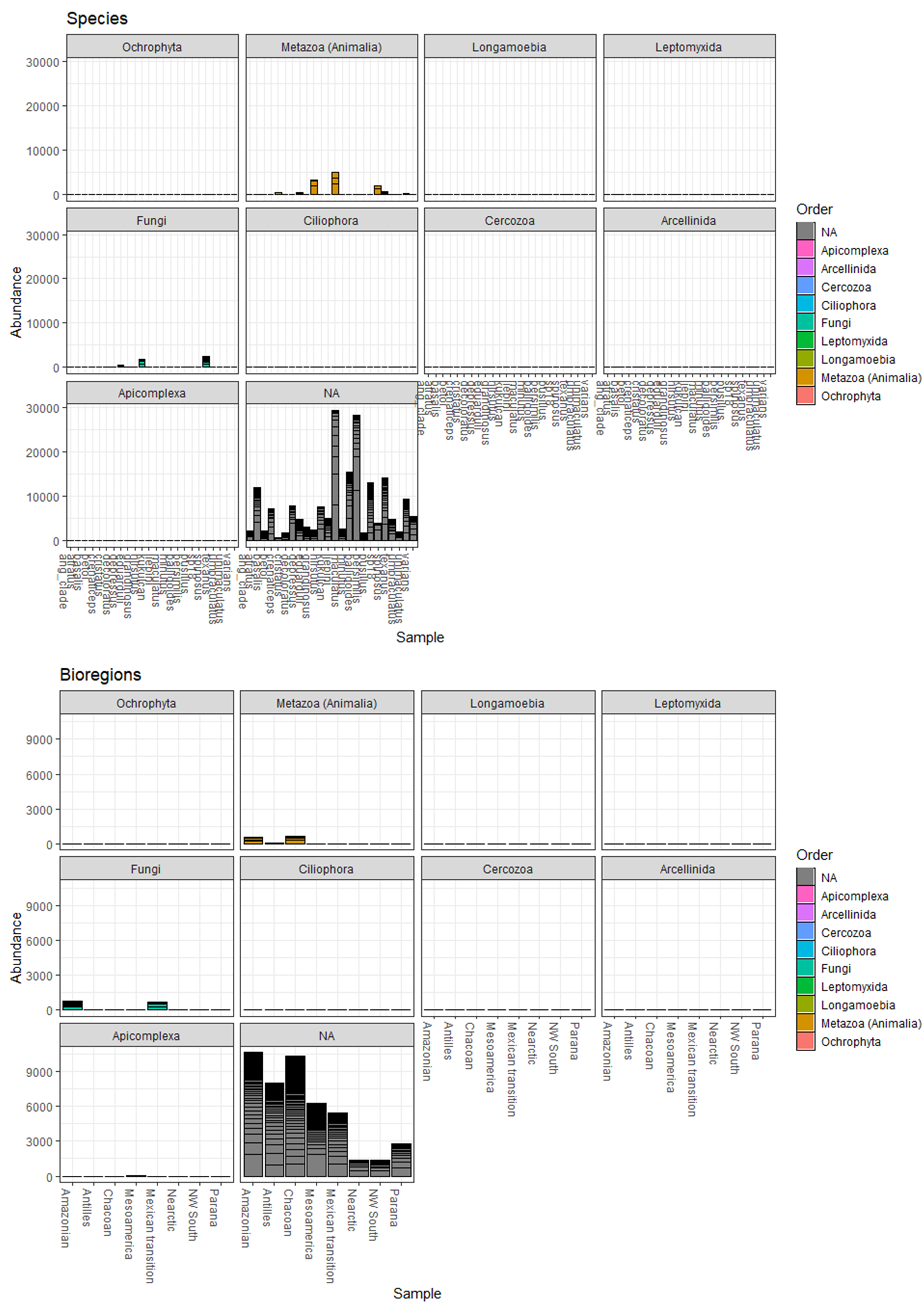

Supplement: Supplementary file 8 — Additional file 8. Bar plots illustrating the abundance of most common Eukaryotic orders associated with turtle ants. A Main orders of ASVs grouped according to different host species of Cephalotes. B Main orders of ASVs grouped according to different biogeographic regions of Cephalotes distribution. [file 42523_2022_223_MOESM8_ESM.tif]

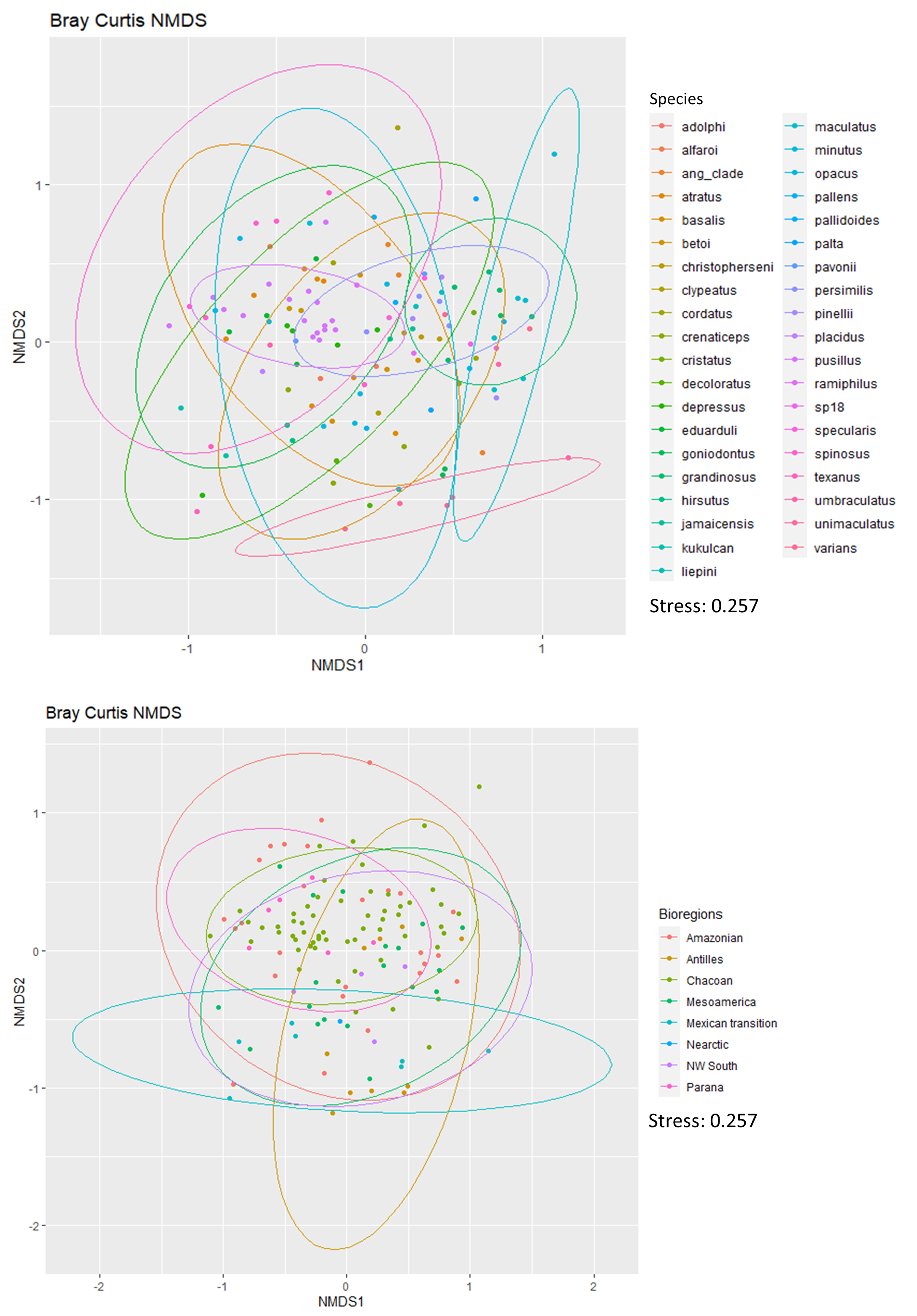

Supplement: Supplementary file 9 — Additional file 9. Non-metric multidimensional scaling (NMDS) (Bray-Curtis) ordination of bacterial communities of Cephalotes samples colored according to different species and biogeographic regions with 95% confidence interval [file 42523_2022_223_MOESM9_ESM.tif]
